# A Matrix-Assisted Laser Desorption/Ionization Time-of-Flight Assay Identifies Nilotinib as an Inhibitor of Inflammation in Acute Myeloid Leukemia

**DOI:** 10.1021/acs.jmedchem.2c00671

**Published:** 2022-09-12

**Authors:** José Luis Marín-Rubio, Rachel E. Peltier-Heap, Maria Emilia Dueñas, Tiaan Heunis, Abeer Dannoura, Joseph Inns, Jonathan Scott, A. John Simpson, Helen J. Blair, Olaf Heidenreich, James M. Allan, Jessica E. Watt, Mathew P. Martin, Barbara Saxty, Matthias Trost

**Affiliations:** † Laboratory for Biological Mass Spectrometry, Biosciences Institute, Newcastle University, Newcastle-upon-Tyne NE2 4HH, UK; ‡ Sir William Dunn School of Pathology, University of Oxford, South Parks Road, Oxford OX1 3RE, UK; § Translational and Clinical Research Institute, Newcastle University, Newcastle-upon-Tyne NE2 4HH, UK; ∥ Respiratory Medicine Unit, Royal Victoria Infirmary, Newcastle upon Tyne Hospitals NHS Foundation Trust, Newcastle upon Tyne NE1 4LP, UK; ⊥ Translational and Clinical Research Institute, Newcastle University, Herschel Building, Level 6, Brewery Lane, Newcastle upon Tyne NE1 7RU, UK; # Newcastle Cancer Centre, Northern Institute for Cancer Research, Medical School, Newcastle University, Paul O’Gorman Building, Framlington Place, Newcastle upon Tyne NE2 4HH, UK; ∇ LifeArc, SBC Open Innovation Campus, Stevenage SG1 2FX, UK

## Abstract

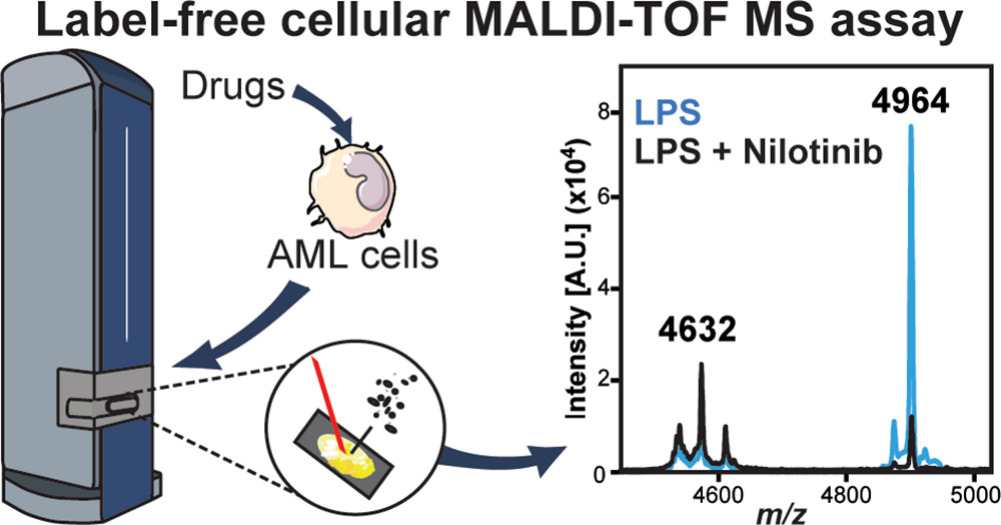

Inflammatory responses are important in cancer, particularly in the context of monocyte-rich aggressive myeloid neoplasm. We developed a label-free cellular phenotypic drug discovery assay to identify anti-inflammatory drugs in human monocytes derived from acute myeloid leukemia (AML), by tracking several features ionizing from only 2500 cells using matrix-assisted laser desorption/ionization-time of flight (MALDI-TOF) mass spectrometry. A proof-of-concept screen showed that the BCR-ABL inhibitor nilotinib, but not the structurally similar imatinib, blocks inflammatory responses. In order to identify the cellular (off-)targets of nilotinib, we performed thermal proteome profiling (TPP). Unlike imatinib, nilotinib and other later-generation BCR-ABL inhibitors bind to p38α and inhibit the p38α-MK2/3 signaling axis, which suppressed pro-inflammatory cytokine expression, cell adhesion, and innate immunity markers in activated monocytes derived from AML. Thus, our study provides a tool for the discovery of new anti-inflammatory drugs, which could contribute to the treatment of inflammation in myeloid neoplasms and other diseases.

## Introduction

Monocyte-derived macrophages play important roles in both physiological and pathological processes. The activation of these cells during pathogen infection or other insults leads, in part, to the pathophysiology of inflammation.^1^ Monocytes can recognize various damage-associated molecular (DAMPs) and pathogen-associated molecular patterns (PAMPs) through specific receptors, such as Toll-like receptors (TLRs). Activation of these receptors leads to the production of cytokines, chemokines, and mediators that are involved in inflammation in myeloid leukemia.^2−4^ Several hematopoietic disorders, including lymphoproliferative disorders and myelodysplastic syndromes, which possess a high risk of transformation to leukemia, have been linked to aberrant TLR signaling.^4−6^ Moreover, TLR2 and TLR4 show significantly higher expression in the bone marrow of patients with myeloid leukemia.^6^ TLR activation leads to intracellular signaling cascades including the mitogen-activated kinase (MAPK) pathways,^7,8^ increasing the expression and secretion of inflammatory cytokines and chemokines including interleukins, interferons, and tumor necrosis factor alpha (TNF-α).^9^ A shared characteristic of many hematologic malignancies is the overproduction of inflammatory cytokines, particularly TNF-α and IL-6 in myeloid malignancies.^4^

The discovery of drugs that prevent chronic or acute inflammation is a major goal of the pharmaceutical industry for the treatment of infectious diseases, autoimmune diseases, and cancer.^10^ The discovery and design of new compounds for inhibiting inflammation are typically achieved through high-throughput screening (HTS) approaches. As such, there is a growing need to develop physiological phenotypic HTS assays that can be used for monocytic cell lines and primary cells to accelerate the discovery of inhibitors.^11,12^ Mass spectrometry (MS)-based readouts in drug discovery have been largely dominated by instruments consisting of solid-phase extraction (SPE) coupled electrospray ionization (ESI; i.e., RapidFire) or surface-based MS techniques, such as matrix-assisted laser/desorption ionization (MALDI).^13^ MALDI time-of-flight (MALDI-TOF) MS is a versatile, label-free technique that has the potential to accelerate HTS of promising drug candidates.^14−16^ MALDI-TOF MS is tolerant to a number of standard buffer components and has rapidly become popular in the field of HTS drug discovery due to its versatility, requiring very small sample quantities, and minimal sample clean-up.^14,15,17−20^ MS-based screening approaches offer the possibility to simultaneously track several molecules in a label-free manner and provide excellent high-quality signals with minimum noise, reproducibility, assay precision, and cost effectiveness when compared to fluorescence-based assays. Furthermore, fluorescence and chemiluminescence methodologies in primary cells and other readouts, such as antibody-based assays, are very expensive, making full-deck screens of millions of compounds difficult.^13,20−22^ MALDI-TOF MS, similar to acoustic mist ionization mass spectrometry,^23^ requires little sample preparation, allows rapid screening, and has high specificity and sensitivity.^13^ Whole cell analyses or cellular assays for evaluating compound efficacy affecting a cellular phenotype present an interesting challenge for MALDI-TOF MS analysis as the system becomes inherently more complex. One of the attractive qualities of this type of assay for the pharmaceutical industry is that the cellular assays provide biologically relevant information about the physiology and general condition of a cell, such as cell viability and protein activity in a single cellular screen.^24,25^ This simultaneous acquisition of information is known as multiplexing and is desirable in cellular assays, as it significantly increases the screening efficiency of the compound library.^26^

In this study, we have developed a cellular MALDI-TOF MS assay that is highly reproducible, robust, and sensitive in different cell lines, with which we can identify two main signatures altered under different pro-inflammatory stimuli. Using this MALDI-TOF MS assay, we performed a blind screen of 96 compounds to assess their potential anti-inflammatory effects on human monocytes derived from acute myeloid leukemia (AML). We discovered that nilotinib, but not the structurally related imatinib, inhibits the pro-inflammatory phenotype. Using thermal proteome profiling (TPP)^27−35^ and whole proteome analyses, we identified direct targets and downstream regulation of nilotinib during inflammation events. Our data showed that nilotinib and other second- and third-generation BCR-ABL inhibitors, but not imatinib, are capable of blocking the p38α MAPK signaling pathway, which abates monocyte activation and differentiation, reduces cytokine release, and prevents inflammation in AML.

## Results

### A Novel MALDI-TOF MS Cellular Assay to Characterize Inflammatory Phenotypes

**Figure 1:**
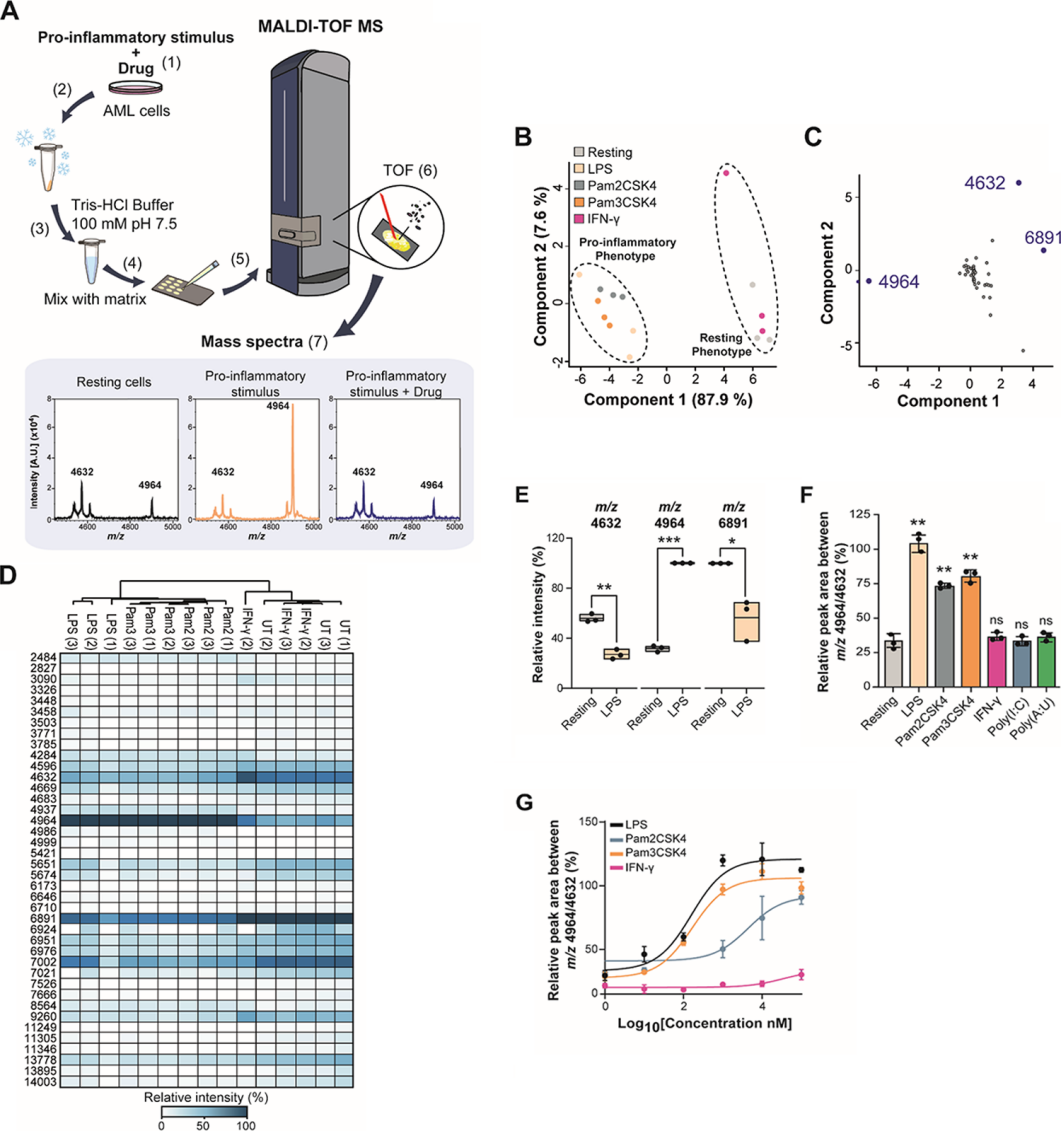
Identification of features associated with the monocyte inflammatory phenotype by MALDI-TOF MS. (A) Workflow of the MALDI-TOF MS assay: (1) AML cells were pre-treated with a drug for 1 h before adding a pro-inflammatory stimulus for 24 h. (2) Cells were frozen on dry ice, thawed, and (3) washed with 100 mM Tris–HCl buffer at 4 °C. (4) Cells were mixed with a matrix (10 mg/mL α-cyano-4-cinnamic acid in 50% acetonitrile, 0.1% trifluoroacetic acid). (5) Cells were analyzed in a rapifleX PharmaPulse MALDI TOF mass spectrometer. (6) In the ionization chamber, a laser is used to produce ions in the gas phase. These ions are separated according to their time-of-flight (TOF) in a field-free region. The smaller ions reach the detector first followed by the bigger ions, according to the *m/z* ratio. (7) The detector converts the received ions into electrical current which is amplified and digitized in *m/z* spectra. (B) Unsupervised PCA plot of LPS, Pam_2_CSK_4_, Pam_3_CSK_4_ and IFN-γ-treated cells showing separation of cells treated with bacterial ligands treated and resting monocytes. (C) Loading plot derived from PCA in panel (B) showing that *m/z* 4964 and 4632 contribute predominantly to the separation of the two clusters in component 1 and component 2. (D) Unsupervised heat map of the relative intensities of three biological replicates of THP-1 cells treated with 100 ng/mL LPS, Pam_2_CSK_4_, Pam_3_CSK_4_, 100 U/mL IFN-γ, 1 μg/mL poly(I:C), and poly(A:U) for 24 h compared to resting cells. (E) Box plots of significantly changing intensities between resting and LPS-treated monocytes identified at *m/z* 4632 and 4964. (F) Relative quantitation from three biological replicates of THP-1 cells treated with 100 ng/mL LPS, Pam_2_CSK_4_, Pam_3_CSK_4_, and 100 U/mL IFN-γ for 24 h compared to resting cells. (G) Titration of LPS, Pam_2_CSK_4_, and Pam_3_CSK_4_-treated cells from 10–100 ng/mL of stimulus. Significant differences between two groups were determined by the Mann–Whitney U-test. The statistical significance of the comparisons with resting is indicated as follows: ns, not significant; ***, *P* ≤ 0.001; **, *P* ≤ 0.01; *, *P* ≤ 0.05. Error bars represent the standard deviation of three biological replicates.

Recently, high-throughput MALDI-TOF MS has been used successfully for *in vitro* assays of specific enzymes.^14−16,36^ Here, we tested if MALDI-TOF MS can be used for phenotypic assays to monitor inflammatory responses by identifying differences in the “fingerprint” of biomolecules ionized when whole cells are spotted onto the MALDI target (Figure 1A). As a proof of concept, we tested if we could distinguish phenotypes of THP-1 cells, a model of human monocytes derived from AML,^37^ upon stimulation with a number of pro-inflammatory stimuli, namely, LPS, Pam_2_CSK_4_, Pam_3_CSK_4_, polyinosinic:polycytidylic acid (poly(I:C)), and polyadenylic–polyuridylic acid (poly(A:U)), which activate TLR4, TLR2/6, TLR1/2, and TLR3, respectively, as well as interferon-γ (IFN-γ). Principal component analysis (PCA) of all biomolecules detected in the range of *m/z* 2000–20,000 showed clear separation of monocytes treated with TLR-agonists, while IFN-γ treatment did not separate from untreated, suggesting that the features are not dependent on interferon stimulation but rather are TLR-dependent (Figure 1B). A loading plot analysis of the PCA results identified the features at *m/z* 4632, 4964, and 6891 as the main drivers for the difference in the phenotypes (Figure 1C). The features at *m/z* 4632 and 6891 were significantly reduced, while *m/z* 4964 increased in LPS-stimulated cells (Figure 1D,E and Figure S1A). As the feature at *m/z* 6891 showed a greater variability between replicates, we selected the features *m/z* 4632 and 4964 for further analysis of the phenotypes. We confirmed that other TLR ligands such as Pam_2_CSK_4_ and Pam_3_CSK_4_, which activate TLR2/6 and TLR1/2, respectively, did also increase this ratio and in a dose-dependent manner, while interferon-gamma (IFN-γ), poly(I:C), and poly(A:U) did not affect it (Figure 1F,G and Figure S1A). Thus, the *m/z* 4632 feature was used as a resting phenotype biomarker, while feature *m/z* 4964 was used as an LPS-stimulated monocyte phenotype biomarker. Together, these results indicate that biomarkers identified by MALDI-TOF MS can be used to detect changes in the inflammatory phenotype downstream of TLRs in AML cells.

### MALDI-TOF MS Cellular Assay Can Detect Inhibitors of Inflammation

**Figure 2:**
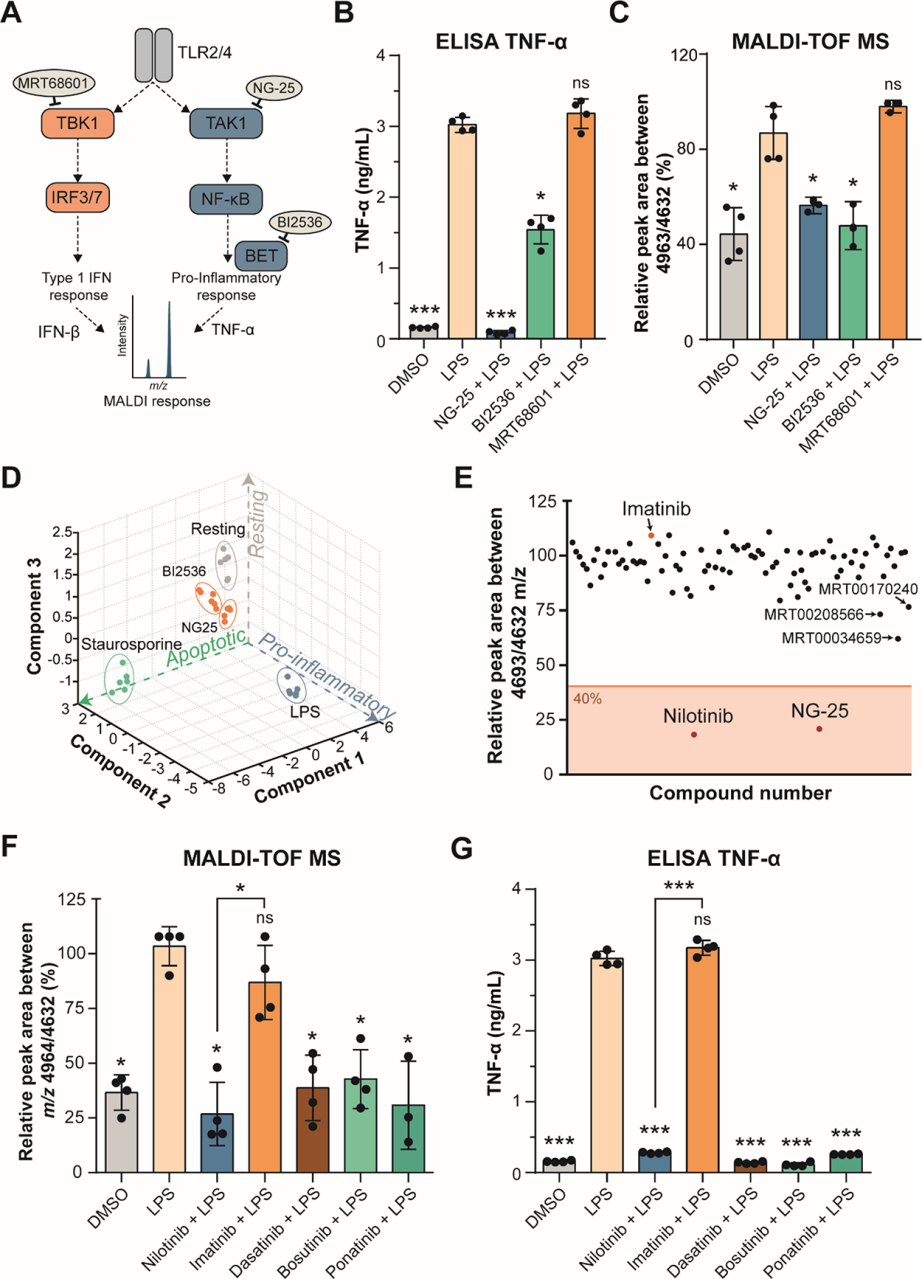
Nilotinib is a positive hit in a cellular MALDI-TOF MS drug discovery screen and inhibits inflammation in response to LPS. (A) Inhibition of the TLR2 and TLR4 signaling pathways. MRT68601 inhibits TBK1 (TANK-binding kinase-1), which blocks production of type I interferons (IFNs). NG-25 inhibits TAK1 (TGF-beta-activated kinase-1), which induces TNF-α and IL-6 production. BI2536 inhibits BET proteins (bromodomain and extra-terminal motif proteins), which are required for pro-inflammatory gene transcription. (B) TNF-α secretion of THP-1 cells treated in either vehicle control-treated (DMSO), 100 ng/mL LPS-treated, or pre-treated with 5 μM NG-25, BI2536 or 1 μM MRT68601 for 1 h before 100 ng/mL LPS treatment for up to 24 h measured by ELISA. (C) MALDI-TOF MS relative quantitation of the normalized ratio *m/z* 4964/4632 in THP-1 cells as treated in panel (B). (D) PCA of all features identified by the MALDI-TOF MS of experiment in panel (B) and 0.5 μM staurosporine-treated cells serving as control for apoptotic cells. (E) Compound hit map of the mean of three biological replicates with a 40% effectiveness cutoff showing nilotinib and NG-25 as positive hits and imatinib as the negative hit (arrows). (F) MALDI-TOF MS relative quantitation of the normalized ratio *m/z* 4964/4632 in THP-1 cells in vehicle control-treated (DMSO), 100 ng/mL LPS-treated, or pre-treated with 5 μM nilotinib, imatinib, 1 μM dasatinib, bosutinib, or ponatinib for 1 h before 100 ng/mL LPS-treatment for up to 24 h. (G) TNF-α secretion of cells treated as in panel (F) measured by ELISA after 24 h. Significant differences between two groups were determined by a Mann–Whitney U-test. The statistical significance of the comparisons with LPS is indicated as follows: ns, not significant; *, *P* ≤ 0.05; **, *P* ≤ 0.01; ***, *P* ≤ 0.001. Error bars represent the standard deviation of four biological replicates.

Next, we used NG-25, a TAK1 inhibitor that blocks TNF-α production, BI2536, a PLK1 inhibitor that has been show to inhibit pro-inflammatory gene transcription due to inhibition of BET proteins,^38^ and MRT68601, a highly selective and potent TBK1 inhibitor that blocks production of type I interferons (IFNs),^39,40^ to test if pharmacological inhibition of these pathways can be detected in our assay (Figure 2A). As LPS provided the greatest response of all inflammatory stimuli (Figure 1F,G), this stimulus was used for further assays. THP-1 cells were pre-treated with the inhibitors for 1 h before LPS-stimulation. NG-25 and BI2536 treatment, but not MRT68601, reduced TNF-α (Figure 2B), IL-6, and IL-1β secretion (Figure S2A,B). It has been published that MRT68601 suppresses the production of type I interferons but increases production of pro-inflammatory cytokines,^39^ which we could not confirm within our experiments. Comparably to the cytokine secretion, our MALDI-TOF MS assay showed that treatment with NG-25 or BI2536 blocked the inflammatory phenotype and was indistinguishable to vehicle control cells not treated with LPS, while MRT68601-treated cells still showed an increased ratio at *m/z* 4964/4632, similar to LPS-treated cells (Figure 2C and Figure S1B). Moreover, using all biomolecule features, PCA analysis showed that treatment with NG-25 reverted the fingerprint back close to control (Figure 2D). To verify that this phenotype was not due to cell death, we performed a cell viability assay (Figure S1L) that confirmed that LPS treatment did not induce apoptosis. Furthermore, cells treated with 0.5 μM staurosporine for 24 h before analysis with our MALDI-TOF assay showed that apoptosis leads to a distinct cluster in the PCA compared to control or LPS-treated cells (Figure 2D**)**, suggesting that this assay can be multiplexed to identify other phenotypes such as cell toxicity. This data shows that pharmacological intervention of inflammatory pathways can be detected by our assay and that NG-25 can be used as a positive control compound in a high-throughput screen.

### Label-Free MALDI-TOF MS Identifies Nilotinib as an Anti-inflammatory Compound

Once the analytical workflow was established to identify an inflammatory phenotype in THP-1 cells, we evaluated the potential of the assay to discriminate positive and negative inhibitors of inflammation in a proof-of-concept blind screen of 96 compounds (Table S1). The assay showed very good sensitivity (*z*′ = ∼0.8) and reproducibility (*R*^2^ > 0.8) (Figure S3A,B). Only two positive hits were observed when applying a cutoff of 40% to the ratio *m/z* 4964/4632, as previously determined for MALDI-TOF MS assays.^15,36^ Unblinding revealed that these compounds were NG-25 and nilotinib (Tasigna) (Figure 2E**)**. Nilotinib is a BCR-ABL tyrosine kinase inhibitor^41,42^ and is a second-generation derivative of imatinib (Gleevec),^42^ which was also included in the panel screened. However, imatinib did not affect the phenotype of inflammatory monocytes, while nilotinib-treated monocytes did not display an LPS-induced inflammatory phenotype and showed no differences compared to nonactivated monocytes in the ratio of *m/z* 4964/4632 (Figure 2F and Figure S1B). Moreover, we tested by MALDI-TOF MS other tyrosine kinase inhibitors (TKIs) used in chronic myeloid leukemia (CML) patients,^43^ the second-generation TKI dasatinib, and the third-generation TKIs bosutinib and ponatinib, which all also reduced the *m/z* 4964/4632 ratio and thus inflammatory responses (Figure 2F and Figure S1C) without affecting cell viability (Figure S1M).

In order to validate the MALDI-TOF MS results, we measured secreted TNF-α levels in THP-1 cells treated with each inhibitor. Nilotinib as well as treatments with the second- and third-generation TKIs reduced secretion levels of TNF-α, IL-6, and IL-1β, while imatinib did not affect TNF-α (Figure 2G), IL-6, and IL-1β levels (Figure S2C,D) upon LPS treatment. Both MALDI-TOF MS and TNF-α ELISA data provided a similar IC50 of approximately 400–500 nM for the inhibitory effect of nilotinib (Figure S3C,D). This data indicates that nilotinib and later-generation TKIs are able to block inflammatory responses downstream of TLR activation, while the structurally similar imatinib cannot.

### Nilotinib Blocks the Inflammatory Response by Inhibiting the p38α MAPK Pathway

**Figure 3:**
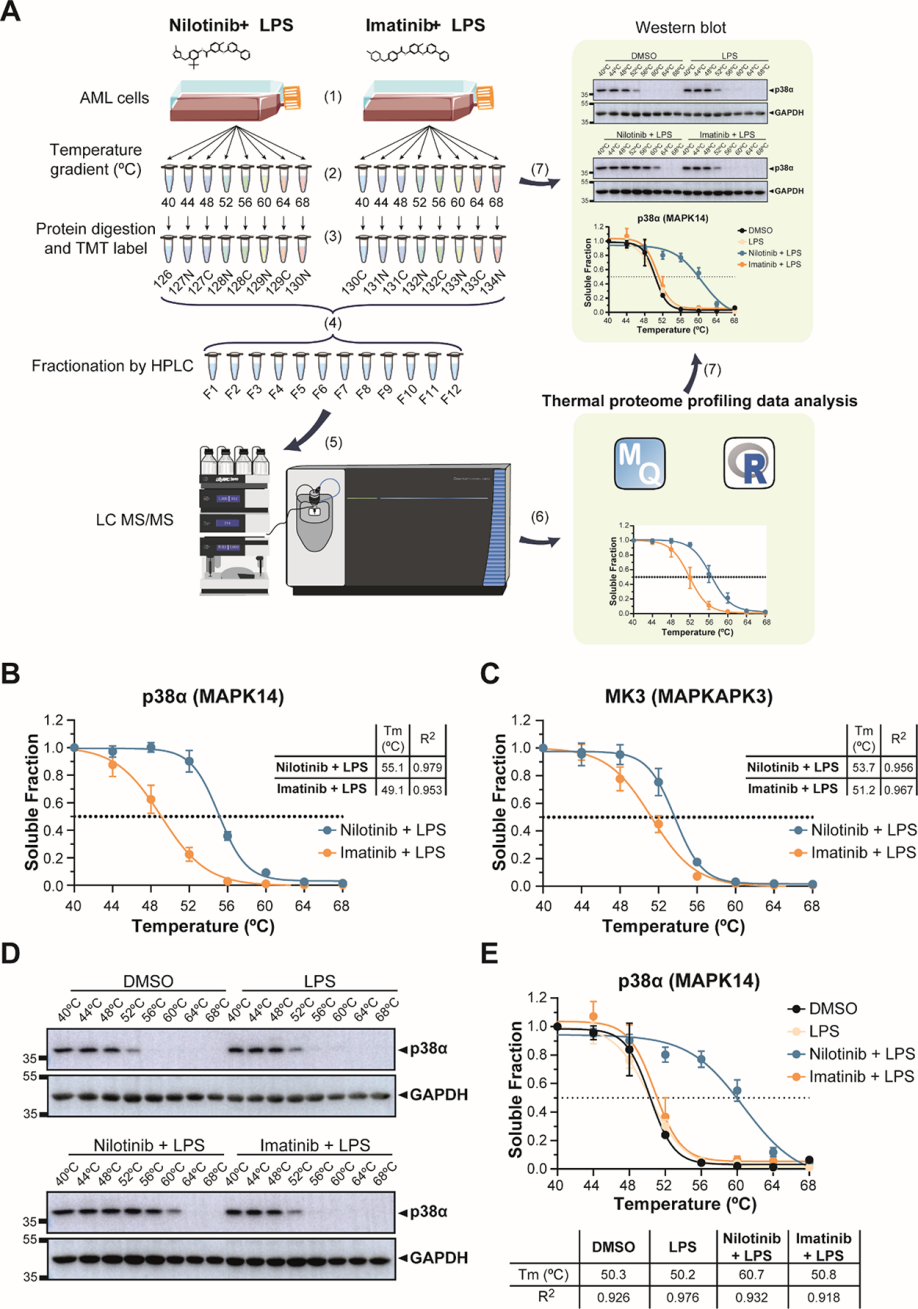
Thermal proteome profiling (TPP) reveals stabilization of p38α and MK3 in nilotinib-treated compared to imatinib-treated cells. (A) Workflow of TPP: (1) AML cells were pre-treated with the drug for 1 h before adding LPS for 15 min. (2) Cells were split in 8 different tubes and heated for 3 min at a range of temperatures (40, 44, 48, 52, 56, 60, 64, and 68 °C). Cells were lysed by freeze-thawing, and precipitates were removed by ultracentrifugation. (3) Proteins in the supernatants were digested with trypsin, labeled with 16-plex tandem mass tags (TMT) and (4) mixed. TMT-labeled peptides were fractionated using high-performance liquid chromatography (HPLC). (5) Peptides were analyzed in an Orbitrap Fusion Lumos Tribrid mass spectrometer. (6) TPP data analysis was performed using MaxQuant and the computing environment R. (7) To validate the results, target proteins were analyzed by western blotting and melting temperatures (*T*_m_) were calculated. (B) Determination of the thermostability of p38α (MAPK14) and (C) MK3 (MAPKAPK3) at the indicated temperatures with 5 μM nilotinib or imatinib for one hour before 100 ng/mL LPS-treatment for up to 15 min by TPP analysis. Table insert shows melting temperature (*T*_m_ °C) and R^2^. (D) Determination of the thermostability of p38α at the indicated temperatures in vehicle control (DMSO), 100 ng/mL LPS and pre-treated with 5 μM nilotinib or imatinib for 1 h before 100 ng/mL LPS-treatment for up to 15 min. GAPDH served as a loading control. (E) Quantification of thermostability of p38α western blots from four independent experiments. Table insert shows melting temperature (*T*_m_ °C) and R^2^. Error bars represent the SEM of four biological replicates. A representative image of four replicates is shown. Relative mobilities of reference proteins (masses in kDa) are shown on the left of each blot.

Nilotinib is known to inhibit BCR-ABL and other tyrosine kinases.^42^ A putative role of these kinases in inflammatory signaling downstream of TLRs is unknown. Moreover, the target involved in this particular function is likely a kinase and must be a specific target for nilotinib since the treatment with imatinib did not result in the same phenotype. In order to identify the off-targets responsible for the nilotinib-specific anti-inflammatory phenotype, we performed thermal proteome profiling (TPP) by multiplexed quantitative mass spectrometry using tandem mass tags (TMT) (Figure 3A, Tables S2 and S3). This method is based on determining the changes in the thermal stability of proteins, which may be due to direct drug binding, drug-induced conformation changes, binding to other cellular components, or post-translational modifications such as phosphorylation.^27,28,31,33,35,44^ We pre-treated THP-1 cells for 1 h with nilotinib or imatinib before stimulation with LPS for 15 min. We reduced the LPS treatment to 15 min in order to avoid changes in protein abundance due to transcriptional responses to LPS activation. Overall, we identified 5565 proteins in our TPP analysis (Table S4 and Figure S4). Only seven proteins changed significantly in all four replicates in melting temperature between nilotinib and imatinib with a standard deviation below two degrees. For three of these proteins, we detected increased Δ*T*_m_ in nilotinib-treated samples. The strongest hit was p38α (MAPK14) with an increased Δ*T*_m_ of 6 °C in nilotinib-treated samples (Figure 3B). The closely related isoform p38γ (MAPK12) did not show significant differences (Figure S5A). Also, MAPK-activated protein kinase 3 (MK3), a downstream protein target of p38, showed an increased Δ*T*_m_ of 2.5 °C in nilotinib-treated samples (Figure 3C). The remainder of significant proteins are shown in Figure S5B–F.

To validate p38α as a target of nilotinib, we performed western blots of lysates from a cellular thermal shift assay. The thermal denaturation temperatures for p38α in DMSO and LPS-treated monocytes were 50.3 and 50.2 °C, respectively. In accordance with the TPP data, the thermal denaturation temperatures for p38α with nilotinib was 60.7 °C, while with imatinib, it was 50.8 °C (Figure 3D,E). Examination of the TPP data for the upstream mitogen-activated protein kinase kinase 3 (MKK3), MKK4, and MKK6 (Figure S5G,J) did not show any differences. We further performed a concentration compound range experiment at 56 °C to determine the amounts of nilotinib required to alter protein thermal stability of p38α. We found that nilotinib was able to stabilize p38α at an EC50 concentration of 4.6 μM (Figure S5K,L). Moreover, we found that nilotinib is also able to affect p38 stability without LPS (Figure S5M,N).

**Figure 4:**
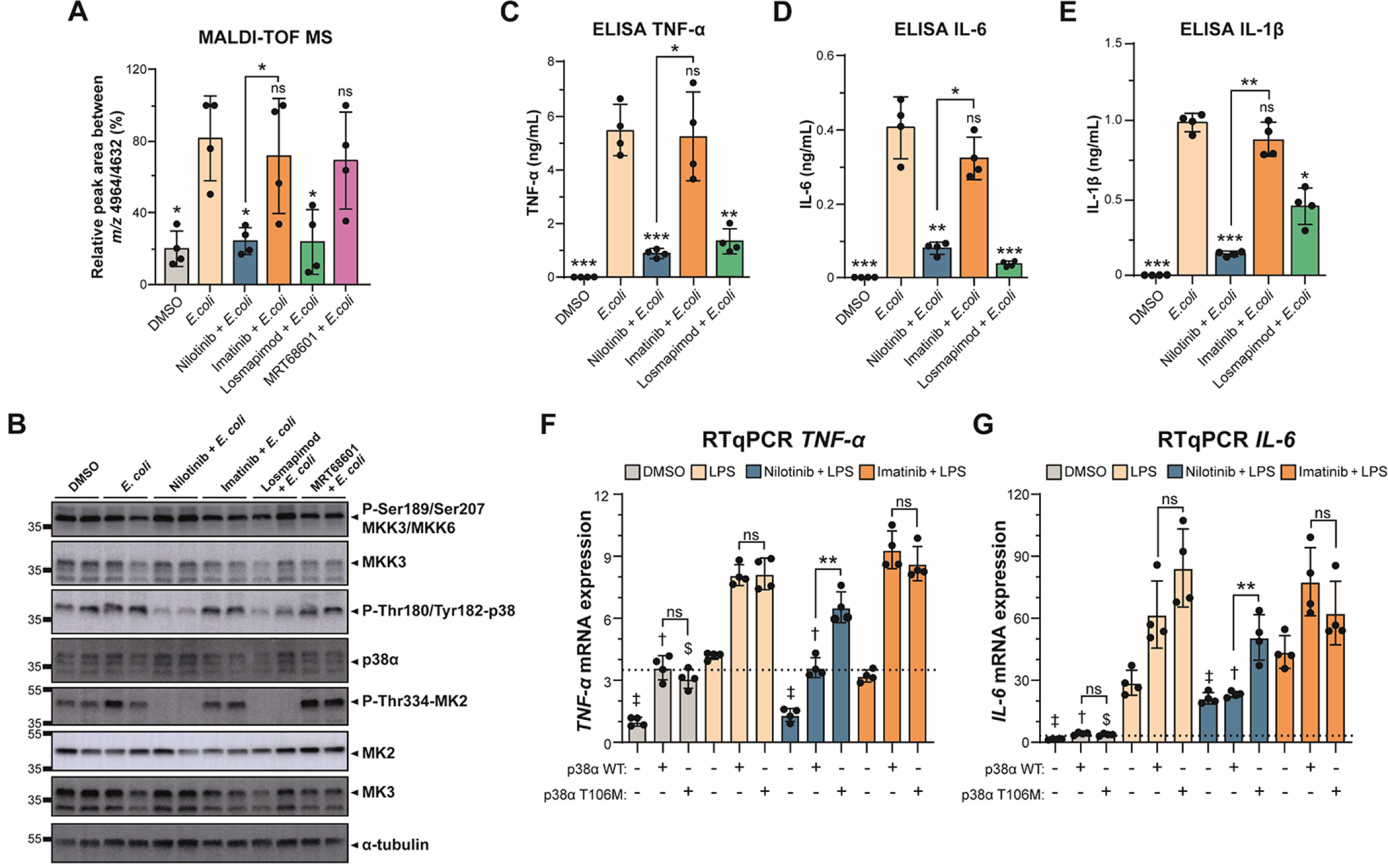
Nilotinib inhibits p38αMAPK-MK2/MK3 signaling axis. (A) MALDI-TOF MS relative quantitation of the ratio *m/z* 4964/4632 in THP-1 cells pre-treated with DMSO, 5 μM nilotinib, imatinib, 1 μM losmapimod, or 1 μM MRT68601 for 1 h before stimulation with live *E. coli* for up to 24 h. (B) Western blot analysis of the p38α MAPK pathway in THP-1 cells pre-treated with DMSO, 5 μM nilotinib, 5 μM imatinib, 1 μM losmapimod, or 1 μM MRT68601 for 1 h before stimulation with live *E. coli* for up to 15 min shows loss of p38 phosphorylation in response to nilotinib. Nilotinib and losmapimod, but not imatinib, block phosphorylation of the downstream MK2. α-Tubulin serves as a loading control. A representative image with two biological replicates of four replicates is shown. Relative mobilities of reference proteins (masses in kDa) are shown on the left of each blot. Relative quantification is shown in Figure S6C. (C) TNF-α, (D) IL-6, and (E) IL-1β secretion was measured by ELISA at 24 h. The statistical significance of the comparisons with *E. coli* is indicated as follows: ns, not significant; **, *P* ≤ 0.01; ***, *P* ≤ 0.001. (F) *TNF-*α and (G) *IL-6* expressions were determined by RT-qPCR in nontransfected or transfected HEK293 cells with p38α WT or p38α M107T for 48 h and pre-treated with DMSO, 5 μM nilotinib, or imatinib for 1 h before stimulation with LPS for up to 24 h. The results were analyzed using the 2^-ΔΔCt^ method and normalized using *GAPDH* and *TBP* as the reference genes and nontransfected DMSO sample as the reference sample. Statistical significance of the comparisons: LPS nontransfected is indicated as ‡; LPS transfected with p38α WT is indicated as †; LPS transfected with p38α T106M is indicated as $. Error bars represent the standard deviation of four biological replicates. The statistical significance of the comparisons between p38α WT and p38α M107T is indicated as follows: ns, not significant; **, *P* ≤ 0.01. Significant differences between two groups were determined by the Mann–Whitney U-test.

To further understand the effect of nilotinib in preventing the inflammatory response by TLR activation in human monocytes, we used *Escherichia coli* (*E. coli*) to stimulate and induce an inflammatory response in THP-1 cells. We used live *E. coli* rather than LPS to activate multiple inflammatory pathways and not only the pathways downstream of TLR4. Similar to LPS treatment, we were able to identify the inflammatory phenotype after exposure to *E. coli* for 24 h using the MALDI-TOF MS assay (Figures S1D and S6A). Moreover, we tested by MALDI-TOF MS the p38 inhibitor losmapimod (GW856553X), which was tested in two phase III clinical trials (ClinicalTrials.gov Identifier: NCT04511819 and NCT02145468). In these results, losmapimod reduced the pro-inflammatory phenotype as well as nilotinib, but not imatinib nor MRT68601 (Figure 4A, Figure S6B, and Figure S1E,F). We observed a reduction in p38α and MK2 phosphorylation in stimulated monocytes treated with nilotinib similar to the specific p38 inhibitor losmapimod (Figure 4B and Figure S6C,D). As nilotinib did not affect the upstream activity of MKK3, it is likely that nilotinib binds p38α directly.

In order to demonstrate that the suppressive effects of nilotinib on the pro-inflammatory phenotype were mediated by inhibition of the p38 MAPK pathway in THP-1 cells, we analyzed the cytokine production mediated by p38 MAPK. In contrast to imatinib, nilotinib significantly reduced TNF-α, IL-6, and IL-1β levels and expression in monocytes stimulated with *E. coli* (Figure 4C,E and Figure S7A–E). To further show that this effect was due to direct binding to p38α, we overexpressed p38α and the drug-resistant p38α T106M.^45^ We found that nilotinib was able to inhibit the effect of p38α but not of the drug-resistant p38α T106M (Figure 4F,G and Figure S7F). Moreover, we performed surface plasmon resonance (SPR) analysis to validate the interaction between p38α and the analyzed TKIs (Figure S8). We found that nilotinib, ponatinib, and dasatinib bind directly to p38α and the affinity constant (*K*_D_) for the interaction was in the low nanomolar range (Figure S8A–C), while bosutinib was in the micromolar range (Figure S8D). Moreover, nilotinib and ponatinib have slow off rates (*K*_off_), which might extend the duration of the compound effect. Imatinib or MRT68601, however, did not bind to p38α (Figure S8E,F). The observed box-shaped sensorgram from the SPR data for bosutinib and imatinib indicates weak or transient protein-drug interactions (Figure S8D,E), as characterized by the rapid association upon sample injection, the steady equilibrium phase, and the rapid dissociation leading back to the buffer baseline. Altogether, our results indicate that nilotinib binds specifically to p38α, which inhibits its activity and affects the production of pro-inflammatory cytokines, thereby reducing inflammation.

### Nilotinib Prevents Monocyte Activation

**Figure 5:**
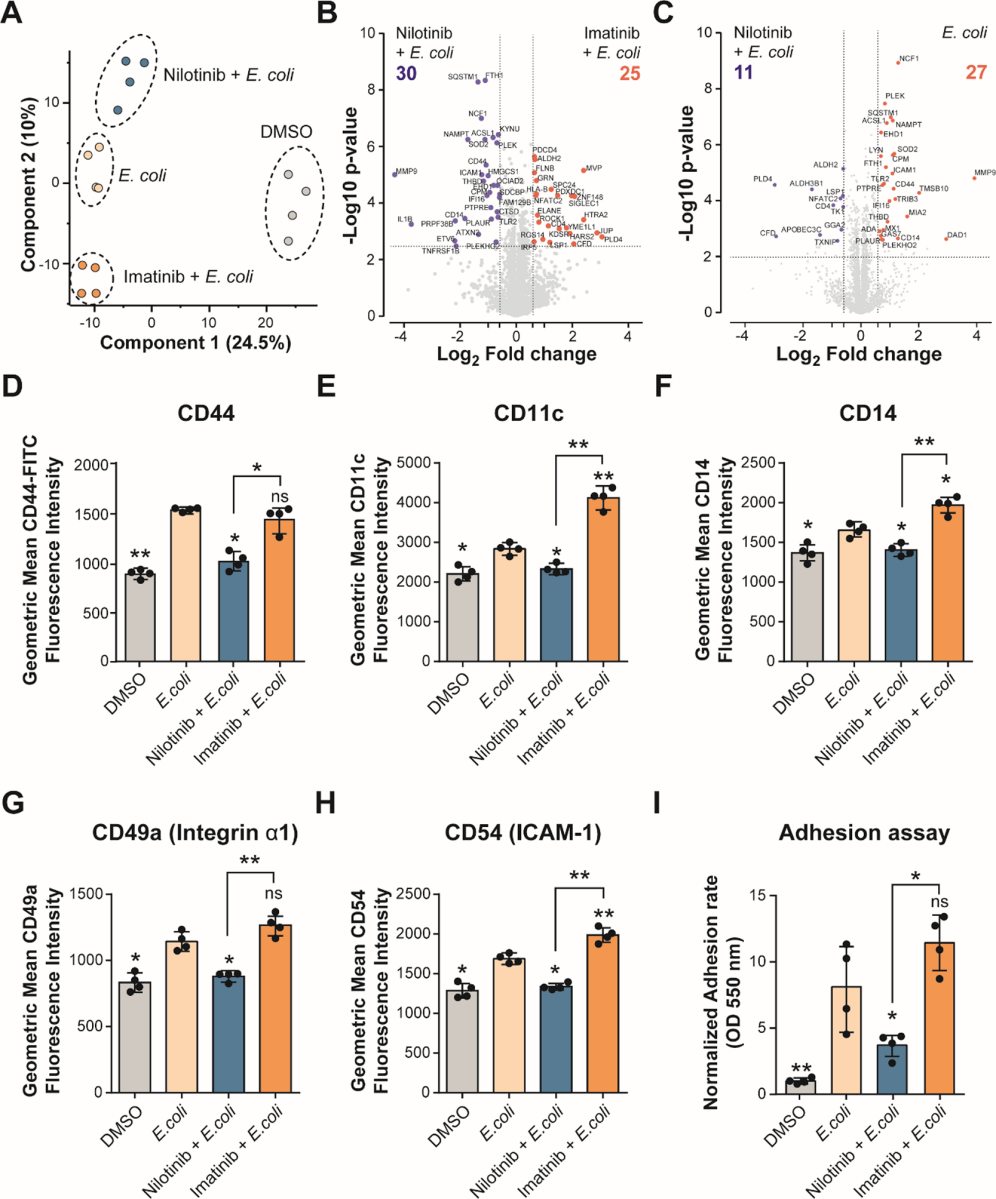
Nilotinib inhibits cell adhesion and monocyte activation. (A) PCA plot of log 2-transformed LFQ intensities from THP-1 cells pre-treated with DMSO, 5 μM nilotinib, or imatinib for 1 h before stimulation with live *E. coli* for up to 24 h, showing distinct grouping of treatments. (B,C) Volcano plot of THP-1 cells treated with (B) nilotinib *vs* imatinib and (C) nilotinib *vsE. coli*, a cutoff of FDR <0.05, and a 1.5-fold change between conditions. (D) Expression levels of CD44, (E) CD11c, (F) CD14, (G) CD49a, and (H) CD54 on the cell surface were measured by flow cytometry. (I) Optical density of dissolved crystal violet was used to evaluate the adhesion rate. Significant differences between two groups were determined by the Mann–Whitney U-test. The statistical significance of the comparisons with *E. coli* is indicated as follows: ns, not significant; *, *P* ≤ 0.05; **, *P* ≤ 0.01. Error bars represent the standard deviation of four biological replicates.

To better understand differences between nilotinib and imatinib treatments on monocytes, we performed quantitative proteomics of THP-1 cells pre-treated with DMSO, 5 μM nilotinib, or imatinib for 1 h before stimulation with live *E. coli* for up to 24 h. We identified and quantified a total of 3570 proteins of which 242 proteins showed significant differences between nilotinib and imatinib treatments (Figure 5A, Table S5, and Figure S8A,B). Comparing differences between nilotinib and imatinib treatments in *E. coli*-stimulated cells, we identified 25 and 30 significant proteins (log 2 fold-change ≤ ±0.6; adjusted *p* value < 0.05) upregulated and downregulated in nilotinib-treated cells compared to imatinib-treated cells, respectively (Figure 5B and Table S6). We found increased levels of proteins related with actin cytoskeleton regulation, such as ROCK1 and Filamin B with nilotinib. Moreover, we found decreased levels of proteins related with cell adhesion, migration, and inflammation response with nilotinib compared to imatinib or *E. coli*-stimulated cells, such as CD14, CD44, ICAM-1, IL-1β, MMP9, and TLR2 (gene ontology and pathway enrichment data in Table S6 and Figure 5B,C). During the inflammatory response, monocyte activation leads to differentiation into macrophages and also leads to an increased expression of cell adhesion proteins.^46−49^

To further understand how nilotinib affected activation of monocytes and their differentiation into macrophages upon stimulation with *E. coli*, we studied cell surface differentiation and cell adhesion markers. We validated our proteomics data by showing that MMP9 and CD44 upregulation induced by *E. coli* was abolished with nilotinib but not with imatinib (Figure S8C and Figure 5D). Furthermore, monocyte activation markers CD11c, CD14, CD49a (integrin α1), and CD54 (ICAM-1) were suppressed with nilotinib treatment but not with imatinib (Figure 5F,H). Also, we observed less cell adhesion with nilotinib treatment than with imatinib (Figure 5I). These results indicate that nilotinib inhibits the inflammatory response of monocytes under a pro-inflammatory stimulus, inhibiting their differentiation into macrophages.

### Nilotinib Reduces the Inflammation Phenotype in AML

Nilotinib is a second-line therapy for CML patients who failed or were intolerant to imatinib,^41,50^ but little is known about its use in other types of cancer. We performed an *in silico* analysis of data from the Genomics of Drug Sensitivity in Cancer (GDSC) database to explore the sensitivity of nilotinib and imatinib sensitivity in different cancer types including hematological and solid tumors. We found that the sensitivity to nilotinib was significantly higher than that to imatinib in hematological malignancies and gastrointestinal tumors (Figure S10A). Interestingly, AML, MM, and diffuse large B cell lymphomas (DLBCL) showed significant differences between the sensitivities to nilotinib and imatinib treatments (Figure S10B). These data support the interest of studying the effects of nilotinib and imatinib in myeloid malignancies as AML. In order to test whether our cellular MALDI-TOF assay can be performed in other cell lines than in THP-1 cells and to test whether nilotinib and other TKIs can be an effective treatment in other monocyte-rich aggressive myeloid neoplasms, we used another monocytic AML cell line, OCI-AML2, and the MM cell line, NCI-H929. We also observed a significant reduction in the ratio *m/z* 4964/4632 by MALDI-TOF MS in both hematopoietic cell lines treated with nilotinib (Figure S10C and Figure S1G,H). Furthermore, we confirmed that nilotinib inhibits p38 MAPK phosphorylation and subsequent MK2 phosphorylation in OCI-AML2 and NCI-H929 cell lines after exposure to *E. coli* (Figure S10D). Moreover, as we observed in THP-1 cells, TNF-α, IL-6, and IL-1β secretion were reduced with nilotinib treatment in OCI-AML2 (Figure S10E).

**Figure 6:**
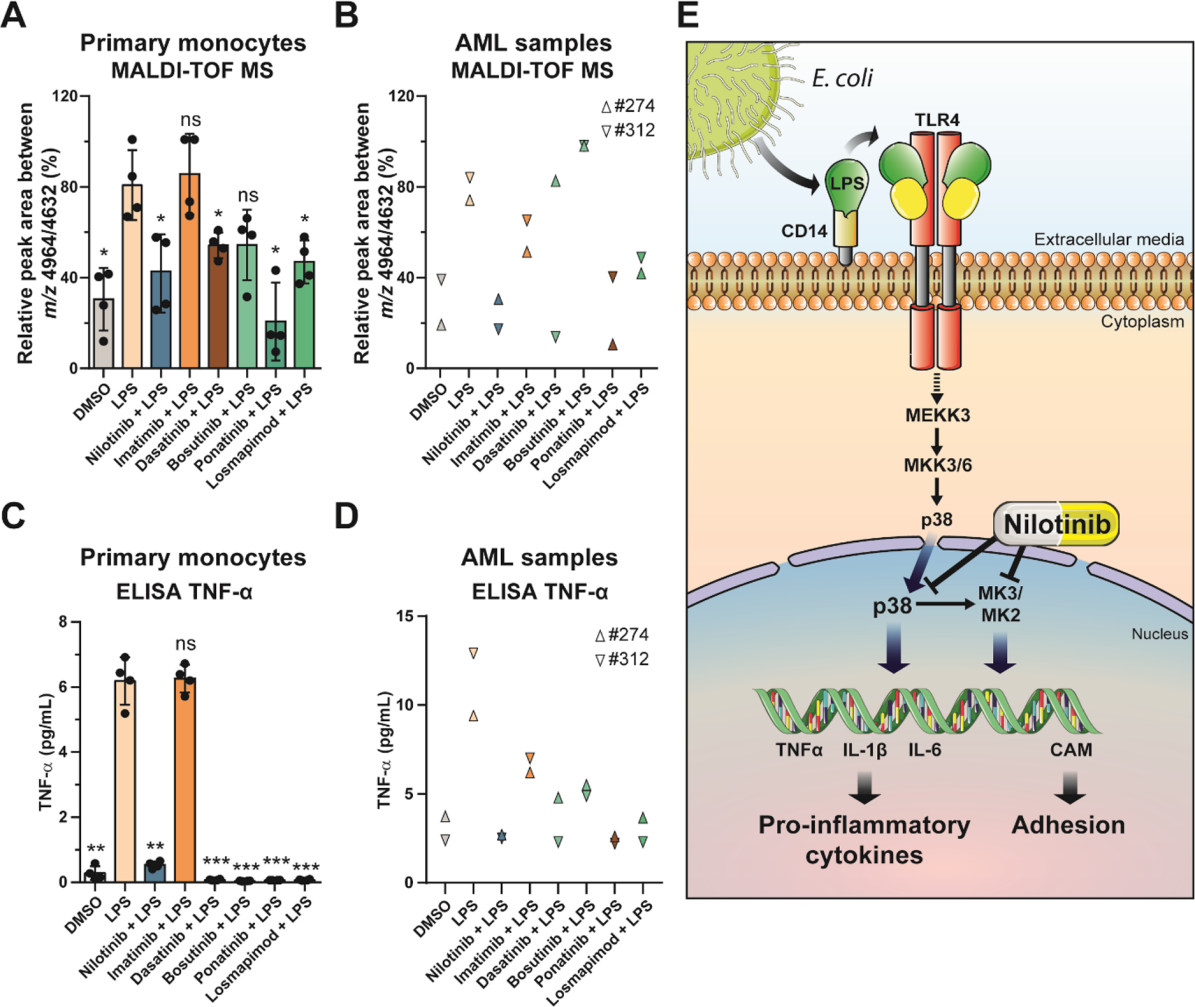
Nilotinib reduces the inflammatory phenotype in AML. (A,B) MALDI-TOF MS relative quantitation of the ratio *m/z* 4964/4632 in (A) human primary monocytes and (B) primary AML cells pre-treated with DMSO, 5 μM nilotinib, imatinib, 1 μM dasatinib, bosutinib, ponatinib, or losmapimod for 1 h before stimulation with 100 ng/mL LPS for up to 24 h. (C,D) TNF-α secretion was measured by ELISA at 24 h in (C) human primary monocytes and (D) primary AML cells. Error bars represent the standard deviation of four or two biological replicates. Significant differences between two groups were determined by the Mann–Whitney U-test. The statistical significance of the comparisons with *E. coli* is indicated as follows: ns, not significant; *, *P* ≤ 0.05; **, *P* ≤ 0.01; ***, *P* ≤ 0.001. (E) Effect of nilotinib during pro-inflammatory stimulation. Activation of TLR4 by a pro-inflammatory stimulus induces p38 MAPK signaling. Nilotinib inhibits p38 phosphorylation and MK2 phosphorylation, reducing the levels of cytokines such as TNF-α, IL-6 or IL-1 β, and cell adhesion molecules (CAMs).

In addition, we performed our MALDI-TOF MS assay in peripheral blood primary human monocytes and primary patient AML cells. For this, we isolated primary human positive CD14 monocytes from four healthy donors and AML cells from two patients (#274 and #312). We also observed a significant reduction in the ratio *m/z* 4964/4632 by MALDI-TOF MS in both LPS-stimulated primary monocytes and AML samples treated with nilotinib as well as with the third-generation TKI, ponatinib, and the p38 inhibitor losmapimod (Figure 6A,B and Figure S1I,J), while ponatinib and bosutinib were more variable. Moreover, TNF-α secretion was reduced with nilotinib treatment in both LPS-stimulated primary monocytes and AML patient cells (Figure 6C,D). Taken together, these results show that our MALDI-TOF MS assay is a suitable tool to detect pro-inflammatory phenotypes via specific biomarkers (*m/z* 4632 and 4964) in AML and monocytes. Furthermore, our data show that nilotinib inhibits the p38α MAPK-MK2/MK3 signaling axis during the inflammatory response, reducing the levels of the pro-inflammatory cytokines in myeloma cells (Figure 6E).

## Discussion

Inflammation has an important role in many aspects of acute myeloid leukemia (AML) such as disease progression, chemoresistance, and myelosuppression.^51^ For the last five decades, AML treatment has been limited to intensive chemotherapy with cytarabine and anthracycline. Nonstandard chemotherapy or immunotherapy is indicated if certain mutations or markers are detected in cancerous cells. Recently, there have been major efforts to understand the disease on the molecular level and to develop novel drugs,^52^ including targeted therapies such as tyrosine kinase inhibitors (TKIs).^53^ To date, 89 drugs targeting protein kinases have been clinically approved by the Food and Drug Administration (FDA, April 2021), while at least 150 are being investigated in clinical trials.^54^ Development of new high-throughput phenotypic drug screening approaches is important for the identification of novel drugs for the treatment of inflammation in autoimmune diseases and cancer. However, current methods for measuring pro-inflammatory cytokines are expensive as they utilize antibodies, making “full deck” analyses of millions of compounds inaccessible.^21,22^ MALDI-TOF MS is a versatile technique with many different applications, ranging from protein identification by peptide mass fingerprinting and small molecule analysis to imaging of tissues.^55,56^ As the technology allows very fast screening, it has already found traction in the HTS field with its application to label-free screening of *in vitro* assays.^13,15,16,36,57−60^

In this study, we present a cheap (Table S7), rapid, and label-free cellular MALDI-TOF MS assay able to identify an inflammatory phenotype in monocytic cells and to screen for anti-inflammatory drugs. We applied a screen to whole cells, allowing the identification of biomarkers that provide a specific fingerprint for the phenotype of the analyzed cells.^61^ Two features at *m/z* 4632 and 4964 were significantly altered upon different inflammatory stimuli, such as, LPS, Pam_2_CSK_4_, Pam_3_CSK_4_, and *E. coli* in the monocytic AML cell line THP-1.^37^ The feature *m/z* 4964 has been previously described as a biomarker to identify patients with Crohn’s Disease, intestinal tuberculosis, or rheumatoid arthritis using MALDI MS imaging.^62,63^ It has been associated with thymosin beta 4 (TB4), a small protein involved in binding and promoting actin polymerization; however, it has also been described as having anti-inflammatory properties.^64^ Since the protein is only 44 amino acids, TB4 was not identified in our TPP or proteomics data and an attempt to identify it by MS/MS directly from cells by MALDI-TOF MS failed; Fourier-transform ion cyclotron resonance mass spectrometry (FT-ICR MS) might allow the identification of these biomarkers. We showed that the cellular MALDI-TOF MS assay can determine the inflammatory phenotype upon activation of the plasma membrane Toll-like receptors (TLR), such as TLR1, TRL2, TLR4, and TLR6, but not upon activation of interferon receptors or the intracellular TLR3 receptor.

While we tested a range of different pro-inflammatory stimuli, we found the greatest response upon LPS stimulation, which was used in a proof-of-concept blind screen of 96 selected compounds. Within this screen, we identified nilotinib as a drug that prevents the inflammatory phenotype in stimulated AML cells by MALDI-TOF MS. However, the structurally similar drug imatinib did not have this anti-inflammatory effect. Imatinib and nilotinib are BRC-ABL tyrosine kinase inhibitors (TKIs) and the standard first-line therapy for CML.^42^ However, nilotinib possesses higher potency than imatinib against BCR-ABL and is active against most imatinib-resistant BCR-ABL mutations.^41^ The second-generation TKIs, dasatinib and bosutinib, as well as the third-generation TKI, ponatinib, are also potent multitargeted tyrosine kinase inhibitors in the treatment of chronic myeloid leukemia.^65^ These compounds have been previously suggested to have potential anti-inflammatory effects, which we confirmed in our data within a range of cell types.^65−68^

Leukemic blasts often secret inflammatory cytokines such as TNF-a, IL-1β, and IL-6, which induce expression of proteins necessary for their adhesion to the vascular endothelium, migration to tissues, proliferation, and chemoresistance.^51,69^ Stimulation with TLR ligands activates monocytes, which leads to the production of cytokines, chemokines, and mediators that are involved in inflammation.^2,70^ TLR ligation induces the formation of a signaling complex that includes IL-1R-associated kinases (IRAKs) and TNFR-associated factor 6 (TRAF6), which mediates the K63-linked polyubiquitylation and activation of TGFβ-activated kinase 1 (TAK1). TAK1 is a MAPK kinase kinase (MAP3K) upstream of p38 MAPK and Jun N-terminal kinases (JNKs). p38 MAPK directly phosphorylates other protein kinases termed MAPK-activated protein kinases (MKs). The MKs that are phosphorylated by and functionally subordinate to p38 MAPK include MK2 and MK3, which play a versatile role in transcriptional and translational regulation, and affect inflammatory responses, triggering the production of cytokines and chemokines.^8,71,72^

Here, we performed a thermal proteome profiling analysis using TMTpro 16-plex,^34^ to identify the possible different targets between nilotinib and imatinib during the inflammatory response. One of the most significant and highly reproducible targets identified was p38α as a main off-target of nilotinib, when compared to imatinib. The direct binding of nilotinib, imatinib, dasatinib, and bosutinib to p38α was described before by an *in vitro* competition binding assay.^73,74^ While this technology is very powerful, TPP analyses detected that nilotinib affected p38α thermal stability but not imatinib. Nilotinib has been reported to inhibit p38α activity^75,76^ and p38γ.^76^ Furthermore, we found MK3 as thermally stabilized by nilotinib, when compared to imatinib, although this could be down to changes in the phosphorylation status. However, we did not find any evidence for p38γ inhibition in our experiments, suggesting remarkable specificity for the p38α isoform. While the loss of p38 phosphorylation would suggest an inhibition upstream of p38 MAPK, we found that no differences for any MAPKK and MAPKK-independent p38 phosphorylation have been described before.^77−79^ Furthermore, our results showed that nilotinib interferes with the downstream MAPK signaling pathway comparable to the specific p38 inhibitor, losmapimod (GW856553X), which has been studied in two phase III clinical trials. Moreover, we found reduction in the pro-inflammatory phenotype, in p38 phosphorylation, and in MK2 phosphorylation when dasatinib, bosutinib, and ponatinib were used. p38α has been described a common off-target for nilotinib, dasatinib, and other TKIs.^73,74,76^

The potential of p38 MAPK inhibitors was initially explored within inflammatory conditions such as cancer, acute myocardial infarction, rheumatoid arthritis, and Crohn’s disease, but the studies demonstrated poor clinical efficacy and unacceptable side effects.^80−84^ This suggests that nilotinib may be considered for additional applications outside of cancer, particularly as it is well tolerated by patients and has few side effects.^41,50^ Within the cancer field, it appears that the anti-inflammatory off-target effects from nilotinib may be beneficial, as seen in tumors co-treated with doxorubicin and vincristine.^85,86^ Consistent with these results, nilotinib, but not imatinib, can reduce the inflammation in human monocytes-derived from AML and other myeloid cells. Moreover, nilotinib and ponatinib had slow dissociation rates (*K*_off_), which has been associated with better selectivity, lower toxicity, and a broader therapeutic window.^87,88^

Dysregulation of TLR signaling has been linked with myeloproliferative disorders.^5,6^ While TLRs are specific for exogenous PAMPs such as lipopolysaccharide (LPS), which triggers TLR4 signaling in monocytes and macrophages, they are also activated by specific endogenous DAMPs such as HSP60 or biglycan.^89,90^ The bacterial surface molecule LPS activates TLR4 and CD14 in monocytes, inducing pro-inflammatory (IL-1, IL-6, and TNF-α) and then anti-inflammatory (IL-10, soluble TNF receptor, and IL-1 receptor antagonist) cytokines.^9,89^ Nilotinib treatment was able to decrease the levels of IL-1β, IL-6, and TNF-α cytokine secretion upon stimulation with *E. coli* in AML cells. In line with our results, pre-incubation with nilotinib in murine macrophages derived from bone marrow (BMDMs) led to a decreased LPS-induced *IL-6* expression.^91^ Furthermore, monocyte differentiation into macrophages induces an upregulation of CD11c and CD14 surface markers^46,47^ as well as cell adhesion molecules.^48,49^ Interestingly, nilotinib was able to block the upregulation of CD11c, CD14, CD44, CD49a, and CD54 markers, thus reducing immune response and cell adhesion of monocytes. We further found MMP9, which cleaves CD44 and induces cell migration and cell–cell and cell–matrix adhesion,^92^ downregulated upon nilotinib treatment. A potential mechanism of action is the nilotinib-dependent increase of ROCK1 levels in *E. coli*-stimulated cells, as deficiency in ROCK1 has been implicated in the migration and recruitment of inflammatory cells such as macrophages and neutrophils during acute inflammation.^93^ Furthermore, we observed a reduction in cell adhesion in activated monocytes upon nilotinib treatment, compared to imatinib. Our data therefore suggests that nilotinib may not only affect inflammatory signaling and immune cell adhesion but also differentiation from monocytes into macrophages.

Our study provides a valuable tool to discover new anti-inflammatory drugs using a cellular MALDI-TOF MS assay, as we demonstrate here for nilotinib. We show that nilotinib inhibits the p38α MAPK-MK2/3 signaling axis and prevents its phosphorylation and subsequent activation, ultimately preventing the transcription of pro-inflammatory genes, cell adhesion markers, and innate immunity markers. In consequence, nilotinib may have therapeutic potential through the inhibition of the p38 MAPK-MK2/3 axis in inflammatory diseases as well as in myeloid malignancies such as myeloid leukemia and multiple myeloma.

## Methods

### Compounds

LPS was purchased from Sigma-Aldrich; Pam_2_CSK_4_, Pam_3_CSK_4_, poly(A:U), and poly(I:C) from Invitrogen; mouse IFN-γ from PeproTech; MRT68601, bosutinib, dasatinib, and ponatinib from Tocris; losmapimod from Biorbyt; and nilotinib, imatinib, NG-25, and the other compounds from Table S1 were provided by LifeArc. All compounds are >95% pure by HPLC analysis.

### Cell Culture

Acute myeloid leukemia-derived cell lines THP-1 (ATCC) and OCI-AML2 (DSMZ), and multiple myeloma-derived cell lines NCI-H929 (ATCC) were cultured in RPMI 1640 medium (Gibco) supplemented with 10% FBS and 4 mM l-glutamine at 37 °C in a humidified 5% CO_2_ atmosphere. ATCC and DSMZ routinely perform cell line authentication, using short tandem repeat profiling as a procedure. Cell experimentation was always performed within a period not exceeding 6 months after resuscitation in mycoplasma-free culture conditions.

### Transient Transfection with Lipofectamine

HEK293 (ATCC) were transfected using the Lipofectamine LTX Reagent and OptiMEM Reduced Serum Medium (Thermo Fisher) with pCMV-MAPK14 (p38α) WT (DU1765) and pCMV-MAPK14 (p38α) T106M (DU3741) from MRC PPU, University of Dundee.

### Isolation of Human Monocytes from Peripheral Blood Mononuclear Cells (PBMCs)

Mononuclear cells were freshly isolated from peripheral blood collected from five healthy donor volunteers. Blood was collected into citrate buffer. PBMC were isolated by density centrifugation using Lymphoprep (Stemcell technologies) according to the manufacturer’s instructions. Monocytes were isolated using the Pan Monocyte Isolation Kit (Miltenyi Biotec) and cultured in RPMI medium supplemented with 10% FBS, 4 mM l-glutamine, and 50 μg/mL penicillin/streptomycin. The study was conducted according to the principles expressed in the Helsinki Declaration, and informed consent was obtained from all participants.

### AML Patient Cells

AML sample cells were collected after informed consent was provided via the Newcastle Hematology Biobank (Ref: 12/NE/0395). The cells were cultured in Iscove’s modified Dulbecco’s medium supplemented with 20% FBS, 4 mM l-glutamine, 50 μg/mL penicillin/streptomycin, 10 ng/mL interleukin-3 (PeproTech), and 20 ng/mL stem cell factor (PeproTech). The study was conducted according to the principles expressed in the Helsinki Declaration, and informed consent was obtained from all participants. #274 sample is positive for FLT3-ITD+ and #312 sample has t(8;21) translocation.

### Blind Drug Screening

One million THP-1 cells/mL were incubated with each compound (Table S1) at 5 μM for 1 h in technical triplicate before stimulation with 100 ng/mL LPS for 24 h. Compounds were staggered into 8 sets each with individual positive (NG-25) and negative controls (MRT68601) to enable maximum relative peak area calculations. This screening process was repeated in three biological replicates.

### Cell Infection with *E. coli*

The DH5α strain of *Escherichia coli* (*E. coli*, Invitrogen) was harvested during the mid-log phase. Mammalian cells were infected with a multiplicity of infection (MOI) of 2. The cells with bacteria were centrifuged at 500*g* for 5 min at 37 °C and incubated at 37 °C in 5% CO_2_ for bacterial uptake for 15 min or 30 min. Thirty minutes post-infection, cells were washed once with PBS and incubated for 1 h with 100 μg/mL gentamicin to kill extracellular bacteria. Then, the cells were washed with PBS twice and the media were replaced with 20 μg/mL gentamicin for the remainder of the experiment.

### Cell Viability Assays

The viability of THP-1 cells was assessed for 24 h using the Cell Proliferation Kit II (XTT) (Sigma-Aldrich) as per manufacturer’s instructions. Following incubation, the plate was read with a SpectraMax iD5 microplate reader (Molecular Devices) at a wavelength of 490 nm. The reference wavelength of 655 nm was also read to control for nonspecific absorption.

### Sample Preparation for MALDI-TOF MS and Data Analysis

Cell pellets were frozen on dry ice, then thawed and washed with 100 mM Tris–HCl, pH 7.5, and centrifuged at 1000*g* for 10 min at 4 °C. Only 2500 cells were spotted on the target with 10 mg/mL α-cyano-4-cinnamic acid in 50% acetonitrile, 0.1% trifluoroacetic acid. Automated target spotting was performed using a Mosquito liquid handling robot (TTP Labtech). A RapifleX PharmaPulse MALDI TOF/TOF mass spectrometer (Bruker Daltonics) equipped with a Smartbeam 3D laser was used in positive ion mode. Samples were acquired in automatic mode (AutoXecute; Bruker Daltonics), totaling 10,000 shots at a 10 kHz frequency per spot. A random walk pattern (complete sample) on a spot laser ablation pattern was used with an M5 Smart beam Parameter at a 45 μm × 45 μm scan range. Spot diameter was limited to 2000 μm and a random walk pattern movement enabled at 1000 shots per raster position. Ionization was achieved using a laser power of 75% (laser attenuator offset 14%, range 30%) with a detector gain of ×6.8 in the mass range of *m/z* 2000–20,000 with a mass suppression up to *m/z* 1600. Samples were analyzed in a linear geometry with optimized voltages for ion sources (ion source 1, 20 kV; pulsed ion extraction 1.3 kV), lens (8.6 kV), and a pulsed ion extraction of 180 ns. A novel 10-bit digitizer was used at a sampling rate of 1.25 GS/s. Raw data were processed first by a TopHat baseline subtraction followed by smoothing with a SavitzkyGolay algorithm. MALDI-TOF spectra were processed by a FlexAnalysis Batch Process (Compass 2.0). Spectra-based PCA plots were generated using ClinPro Tools (Bruker Daltonics). A normalized relative peak area was used to measure a pro-inflammatory response between *m/z* 4632 and 4963 and was calculated with the following equation:
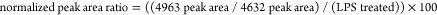


This method of intraspectra quantification^94^ was robust over 10 different passages, where we were able to quantify the inflammatory response by MALDI-TOF MS with *Z*′ > 0.5 and *P* ≤ 0.001.

### Quantitative RT-PCR

RNA was extracted using Trizol (Thermo Fisher Scientific). Real-time RT-qPCR from total RNA in two steps was performed with a QuantiTect reverse transcription kit (Qiagen) and a QuantiTect SYBR green kit (Qiagen) using a StepOne Applied Biosystems real-time PCR system (Thermo Fisher Scientific). Expression values of *GAPDH* and *TBP* genes in the same samples were used for normalization, using the 2^-ΔΔCT^ method. The following primers were used: *GAPDH*, forward: GTCTCCTCTGACTTCAACAGCG and reverse: ACCACCCTGTTGCTGTAGCCAA; *IL-6*, forward: GCCCAGCTATGAACTCCTTCT and reverse: CTTCTCCTGGGGGTACTGG; *TBP*, forward: GAGTTCCAGCGCAAGGGTTT, and reverse: GGGTCAGTCCAGTGCCATA; *TNF-*α, forward: AAACTCATGAGCAGTCTGCA and reverse: AGGAGATCTTCAGTTTCGGAGG.

### ELISA

The cell culture supernatant was collected at 24 h post-treatment. TNF-a, IL-6, and IL-1β were measured by DuoSet ELISA kits (R&D Systems). Absorbance from four biological replicates at 450 nm was measured with the correction wavelength set at 540 nm using a SpectraMax M3 microplate reader (Molecular Devices).

### Western Blot

Cells were lysed using 5% SDS supplemented with the Protease Inhibitor Cocktail and Phosphatase Inhibitor Cocktail 2 (Sigma-Aldrich). The following antibodies were purchased from Cell Signaling Technology (Danvers): MK2 (#3042), Thr-334-P-MK2 (#3041), MK3 (#7421), MKK3 (#8535), Ser189-P-MKK3/Ser207-P-MKK6 (#12280), p38 MAPK (#9212), p38α MAPK (#9218), p38γ MAPK (#2307), Thr180/Tyr182-P-p38 MAPK (#4511), MPP9 (#13667), anti-rabbit IgG-HRP (#7074), and anti-mouse IgG (#7076). GAPDH (sc-47724) was from Santa Cruz, and α-tubulin (T9026) was from Sigma-Aldrich. The Amersham Imager 600 digital imaging system (GE Healthcare) was used for image acquisition.

### Flow Cytometry

Cell surface staining was performed by the direct immunofluorescence assay with fluorescent-conjugated antibodies: CD11c-APC (#17-0114-82), CD14-AF700 (#56-0149-42), and CD54-FITC (#11-0541-82) from Thermo Fisher; CD49a-PE (#562115) and CD44-FITC (#338803) from BD Biosciences; and corresponding isotype control antibodies, for 30 min at 4 °C in PBS with 1% FBS, 1% BSA, and 1% human serum (Sigma) to block Fc receptors. Cells were analyzed in a FACSCanto II flow cytometer (Becton-Dickinson). The results were analyzed using FlowJo.

### Adhesion Assay

Cells were washed twice with PBS before being fixed with 4% paraformaldehyde for 15 min. Then, cells were incubated with 5 mg/mL crystal violet for 10 min before lysis with 2% SDS for 30 min. Absorbance from four biological replicates at 550 nm was measured using a SpectraMax M3 microplate reader (Molecular Devices). Micrographs were acquired with an inverted microscope Axio Vert.A1 FL LED (Zeiss, Cambridge).

### Surface Plasmon Resonance (SPR)

SPR ligand interactions assays were performed on a Biacore S200 (Cytiva Life Sciences) at 2 °C using multicycle settings. Biotinylated avidin-p38α protein (MRC-Reagents, Dundee) was immobilized onto a Streptavidin surface chip, through injection of 50 μg/mL p38α in DMSO-free SPR running buffer (20 mM HEPES, 150 mM NaCl, 0.1 mM EGTA, 0.5 mM TCEP, 0.01% Tween-20, pH 7.4) over the active flow cell eliciting final captured response units (RUs) of 7719 RUs. The inhibitor analytes (20 mM HEPES, 150 mM NaCl, 0.1 mM EGTA, 0.5 mM TCEP, 0.01% Tween-20, pH 7.4, 1% DMSO) were then injected over both control and active surfaces for 90 s at 30 μL/min before being allowed to dissociate for 360 s (imatinib, dasatinib, bosutinib, and MRT68601) and 600 s (nilotinib, ponatinib) over 10 concentration series to record dose–responses: 0.05–333.33 nM for nilotinib and ponatinib, 0.51–10 μM for dasatinib and bosutinib, and 2.54–50 μM for imatinib and MRT68601. A solvent correction was applied to the data collection, and an 8-point DMSO solvent correction was applied. Responses were analyzed using Biacore Evaluation Software (Cytiva Life Sciences) using affinity fit to determine the *K*_d_. Data are representative of three technical replicates.

### Cellular Thermal Shift Assay

Cells were treated with 5 μM nilotinib or imatinib for 1 h, and then 100 ng/mL LPS was added for 15 min. Cells were washed with PBS supplemented with cOmplete, EDTA-free Protease Inhibitor Cocktail (Sigma-Aldrich), 1.2 mM sodium molybdate, 1 mM sodium orthovanadate, 4 mM sodium tartrate dihydrate, 5 mM glycerophosphate, and 20 mM *N*-ethylmaleimide, which were then separated into 8 fractions for thermal profiling. Fractions were heated at 40, 44, 48, 52, 56, 60, 64, and 68 °C for 3 min. For concentration compound range experiments, cells were treated with 0.1, 0.5, 1, 5, or 10 μM nilotinib or imatinib for 1 h before treatment with 100 ng/mL LPS for 15 min. Cells were heated at 56 °C for 3 min. Samples were lysed with four freeze–thaw cycles using dry ice and a thermoblock at 35 °C. Cell lysates were centrifuged at 4000*g* for 2 h at 4 °C to separate protein aggregates from soluble proteins. Supernatants were collected and used for western blotting and mass spectrometry.

### Sample Preparation for LC–MS/MS and Label-Free Quantification (LFQ) Data Analysis

Samples were prepared for mass spectrometry using S-Trap micro columns (Protifi) according to the manufacturer’s recommended protocol. Proteins were reduced with 20 mM TCEP at 37 °C for 30 min and alkylated with 20 mM *N*-ethylmaleimide in the dark for 30 min. A ratio of 1:10 (w:w) trypsin TPCK treated (Worthington-Biochem) was used to digest the samples for 2 h at 47 °C. Eluted peptides were dried down and resuspended in loading buffer (2% acetonitrile, 0.1% trifluoroacetic acid). Peptide samples were injected on a Dionex Ultimate 3000 RSLC (Thermo Fisher Scientific) connected to an Orbitrap Fusion Lumos Tribrid mass spectrometer (Thermo Fisher Scientific). Samples were injected on a PepMap 100 C18 LC trap column (300 μm ID × 5 mm, 5 μm, 100 Å) followed by separation on an EASY-Spray column (50 cm × 75 μm ID, PepMap C18, 2 μm, 100 Å) (Thermo Fisher Scientific). Buffer A consisted of water containing 0.1% FA and Buffer B of 80% acetonitrile containing 0.1% FA. Peptides were separated with a linear gradient of 3–35% Buffer B over 180 min followed by a step from 35–90% Buffer B in 0.5 min at 250 nL/min and held at 90% for 4 min. The gradient was then decreased to 3% Buffer B in 0.5 min at 250 nL/min, and the column was equilibrated for 10 min before the next injection. The column temperature was controlled at 45 °C. The Orbitrap Fusion Lumos Tribrid mass spectrometer was operated in data-dependent, positive ion mode. Full scan spectra were acquired in the range of *m/z* 400 to 1600 at a resolution of 120,000 with an automatic gain control (AGC) target of 4 × 10^5^ and a maximum injection time of 50 ms. The Orbitrap was operated in ‘stop speed’ to maintain a 3 s fixed duty cycle. The most intense precursor ions were isolated with a quadrupole mass filter width of 1.6 *m/z*, and higher-energy collision-induced dissociation (HCD) fragmentation was performed in a one-step collision energy of 30%. Detection of MS/MS fragments was acquired in the linear ion trap in rapid scan mode with an AGC target of 1 × 10^4^ ions and a maximum injection time of 45 ms. An electrospray voltage of 2.0 kV and capillary temperature of 275 °C, with no sheath and auxiliary gas flow, were used.

All discovery proteomics RAW mass spectra were analyzed using MaxQuant (version 1.6.10.43)^95^ and searched against a SwissProt *Homo sapiens* database (containing 42,347 database entries with isoforms; downloaded on 21 April 2020). Trypsin/P was set as the proteolytic enzyme, *N*-ethylmaleimide on cysteine was set as the fixed modification, and methionine oxidation and acetylation of protein N-termini was set as variable modifications. Two missed cleavages were allowed. A protein and peptide false discovery rate (FDR) of less than 1% was employed in MaxQuant. LFQ data analysis was performed using Perseus (version 1.6.2.3).^96^ Reverse hits, contaminants, and proteins only identified by the site were removed before downstream statistical and bioinformatics analysis. LFQ intensity data were transformed (log 2) and filtered to contain at least two unique peptides and at least three valid values in one group for comparisons. Significance testing was carried out using a two-tailed unpaired Student’s *t* test and multiple hypothesis testing was controlled using the Benjamini–Hochberg FDR threshold of *p* value <0.05. Gene ontology term enrichments were performed within DAVID (version 6.8).^97^

### Thermal Proteome Profiling (TPP) and Data Analysis

Isobaric labeling of peptides was performed using the 16-plex tandem mass tag (TMT) reagents (Thermo Fisher Scientific) (Table S2), according to the manufacturer recommended protocol. According to the different temperature points, labeled peptides were combined and desalted with C18 Macro Spin Columns (Harvard Apparatus, Holliston, MA, USA). The TMT-labeled samples were subjected to fractionation using basic-pH reversed-phase liquid chromatography on a Dionex Ultimate 3000 HPLC system (Thermo Fisher Scientific) using a Gemini C18 column (250 mm × 3 mm, 3 μm, 110 Å; Phenomenex). Buffer A consisted of 20 mM ammonium formate pH 8.0 and Buffer B of 100% acetonitrile. Peptides were fractionated using a linear gradient of 1–49% Buffer B over 49 min at 250 nL/min. Twelve fractions were dried under vacuum centrifugation and resuspended in 2% acetonitrile with 0.1% TFA for LC–MS/MS analysis. Labeled peptide samples were injected on a Dionex Ultimate 3000 RSLC (Thermo Fisher Scientific) connected to an Orbitrap Fusion Lumos Tribrid mass spectrometer (Thermo Fisher Scientific). Buffer A consisted of water containing 0.1% FA and Buffer B of 80% acetonitrile containing 0.1% FA. Peptides were separated with a linear gradient of 3–35% Buffer B over 180 min followed by a step from 35–90% Buffer B in 0.5 min at 250 nL/min and held at 90% for 4 min. The gradient was then decreased to 3% Buffer B in 0.5 min at 250 nL/min, and the column was equilibrated for 10 min before the next injection. The scan sequence began with an MS1 spectrum (Orbitrap analysis; resolution 120,000; mass range *m/z* 375–1500; automatic gain control (AGC) target 4 × 10^5^; maximum injection time 50 ms). MS2 analysis consisted of collision-induced dissociation (CID); AGC 1 × 10^4^; normalized collision energy (NCE) of 30%; maximum injection time of 50 ms; and isolation window of 0.7. Following acquisition of each MS2 spectrum, MS3 precursors were fragmented by high energy collision-induced dissociation (HCD) and analyzed using the Orbitrap (NCE 55%; AGC 5 × 10^4^; maximum injection time 86 ms, resolution was 50,000) and synchronous precursor selection (SPS) was enabled to include 10 MS/MS fragment ions in the FTMS3 scan.

Mass spectrometry data analysis was performed similarly as described above, but TMT modification on the peptide N-termini or lysine residues was enabled for the 16-plex TMT reagents, and deamidation of asparagine and glutamine was included as variable modification in MaxQuant. TPP data analysis was performed using the TPP packages (https://www.bioconductor.org/packages/release/bioc/html/TPP.html) in the R statistical programming language. The data were normalized^35^ (Table S3). Proteins that changed significantly in the four replicates between nilotinib and imatinib with a standard deviation below two degrees in the melting temperature were selected as positive hits.

### Data Availability

The mass spectrometry proteomics data have been deposited to the ProteomeXchange Consortium^98^ via the PRIDE partner repository^99^ with the data set identifier: PXD025020.

### Statistical Analysis

The Shapiro–Wilk test was used to check expression data sets for normality, and the Levene test was used for homogeneity of variances. The Mann–Whitney U-test was performed in all the analysis, excluding proteomics and thermal proteome profiling.

## Supporting Information Available

Figure S1: MALDI-TOF MS spectra; Figure S2: Nilotinib reduces IL-6 and IL-1β secretion; Figure S3: MALDI-TOF MS screening quality control; Figure S4: TPP ratio heatmap; Figure S5: TPP analysis; Figure S6: Nilotinib reduces the pro-inflammatory phenotype; Figure S7: Nilotinib inhibits the pro-inflammatory phenotype in *E. coli*-activated monocytes; Figure S8: Assessment of TKIs against p38α using surface plasmon resonance (SPR); Figure S9: Differences in the proteome of nilotinib- and imatinib-treated monocytes; Figure S10: Nilotinib and imatinib show different sensitivity in blood malignancies (PDF)
Table S1: List of compounds used in MALDI-TOF MS screen; Table S2: TMT labels; Table S3: Proteins used to normalize TPP data; Table S4: TPP analysis; Table S5: Proteomics ANOVA analysis; Table S6: Proteomics Limma analysis and Gene ontology data; Table S7: MALDI-TOF MS, RTqPCR and ELISA prices for 96 drugs discovery assay and per sample (XLSX)

## Abbreviations Used

AML: acute myeloid leukemia
BET: bromodomain and extra-terminal motif proteins
CAMs: cell adhesion molecules
CML: chronic myeloid leukemia
DAMPs: damage-associated molecular
EC50: half maximal effective concentration
ESI: electrospray ionization
FDR: false discovery rate
FDA: Food and Drug Administration
GDSC: genomics of drug sensitivity in cancer
HPLC: high-performance liquid chromatography
HTS: high-throughput screening
IC50: half-maximal inhibitory concentration
IL: interleukin
IRAKs: IL-1R-associated kinases
IFN: interferons
JNK: jun N-terminal kinases
LPS: lipopolysaccharides
*m/z*: mass-to-charge ratio
MALDI-TOF: matrix-assisted laser/desorption ionization-time of flight
MAPK: mitogen-activated kinase
MK: MAPK-activated protein kinase
PAMPs: pathogen-associated molecular patterns
poly(A:U): polyadenylic-polyuridylic acid
poly(I:C): polyinosinic:polycytidylic acid
PCA: principal component analysis
SPE: solid-phase extraction
SPR: surface plasmon resonance
TAK1: TGF-beta-activated kinase-1
TMT: tandem mass tags
TBK1: TANK-binding kinase-1
TPP: thermal proteome profiling
TB4: thymosin beta 4
TKIs: tyrosine kinase inhibitors
TRAF6: TNFR-associated factor 6
TLRs: Toll-like receptors
TNF-α: tumor necrosis factor alpha
