# Supplementary material for: A Matrix-Assisted Laser Desorption/Ionization Time-of-Flight Assay Identifies Nilotinib as an Inhibitor of Inflammation in Acute Myeloid Leukemia
Source: J Med Chem. 2022 Sep 12;65(18):12014–30. doi: 10.1021/acs.jmedchem.2c00671 (PMC9511480; doi:10.1021/acs.jmedchem.2c00671)
Supplement: Supplementary file 1 — jm2c00671_si_001.pdf [file jm2c00671_si_001.pdf]

Supporting Information

A MALDI-TOF assay identifies nilotinib as an inhibitor of inflammation in acute myeloid leukemia

José Luis Marín-Rubio[1,#,*], Rachel E. Peltier-Heap[1,#], Maria Emilia Dueñas[1], Tiaan Heunis[1,2], Abeer Dannoura[1], Joseph Inns[1], Jonathan Scott[3], A. John Simpson[3,4], Helen J. Blair[5], Olaf Heidenreich[5], James M. Allan[5], Jessica E. Watt[6], Mathew P. Martin[6], Barbara Saxty[7], and Matthias Trost[1,*]

[1] Laboratory for Biological Mass Spectrometry, Biosciences Institute, Newcastle University, Newcastle-upon-Tyne, NE2 4HH, UK.

[2] Sir William Dunn School of Pathology, University of Oxford, South Parks Road, Oxford OX1 3RE, UK.

[3] Translational and Clinical Research Institute, Newcastle University, Newcastle-upon-Tyne, NE2 4HH, UK.

[4] Respiratory Medicine Unit, Royal Victoria Infirmary, Newcastle upon Tyne Hospitals NHS Foundation Trust, Newcastle upon Tyne, NE1 4LP, UK.

[5] Translational and Clinical Research Institute, Newcastle University, Herschel Building, Level 6, Brewery Lane, Newcastle upon Tyne NE1 7RU, UK.

[6] Newcastle Cancer Centre, Northern Institute for Cancer Research, Medical School, Newcastle University, Paul O'Gorman Building, Framlington Place, Newcastle upon Tyne NE2 4HH, UK.

[7] LifeArc, SBC Open Innovation Campus, Stevenage, SG1 2FX, UK.

[#] These authors contributed equally to this work.

* Corresponding authors:

José Luis Marín-Rubio. Tel: +44 191 2088983. E-mail: jose.marin-rubio@newcastle.ac.uk

Matthias Trost, Tel: +44 191 2087009. E-mail: matthias.trost@ncl.ac.uk

CONTENT: Pages

Figure S1. MALDI-TOF MS spectra…… S3

Figure S2. Nilotinib reduces IL-6 and IL-1β secretion…… S7

Figure S3. MALDI-TOF MS screening quality control…… S8

Figure S4. TPP ratio heatmap…… S9

Figure S5. TPP analysis…… S10

Figure S6. Nilotinib reduces the pro-inflammatory phenotype…… S12

Figure S7. Nilotinib inhibits the pro-inflammatory phenotype in *E. coli*-activated monocytes…… S13

Figure S8. Assessment of TKIs against p38α using surface plasmon resonance (SPR)… S15

Figure S9. Differences in the proteome of nilotinib- and imatinib-treated monocytes….. **S16**

Figure S10. Nilotinib and imatinib show different sensitivity in blood malignancies. Nilotinib reduces the inflammation phenotype in OCI-AML2 and NCI-H929 cell line…………… **S18**

Table S1. List of compounds used in MALDI-TOF MS screen. The table indicates the drug name, CHEMBL ID, and molecular formula.

Table S2. TMT labels. The table indicates the sample name, TMT label reagent, and HCD monoisotopic report mass (Da).

Table S3. Proteins used to normalize TPP data. Document indicates description and data.

Table S4. TPP analysis. Document indicates description and data.

Table S5. Proteomics ANOVA analysis. Document indicates description and data.

Table S6. Proteomics Limma analysis and Gene ontology data. Document indicates description, data, downregulated and upregulated gene ontology and KEGG enrichment analyses.

Table S7. MALDI-TOF MS, RTqPCR and ELISA prices for 96 drugs discovery assay and per sample. The table indicates the techniques (MALDI-TOF MS, RTqPCR and ELISA), company and reference number, price and units, price for 96 samples and price per sample.

Figure S1

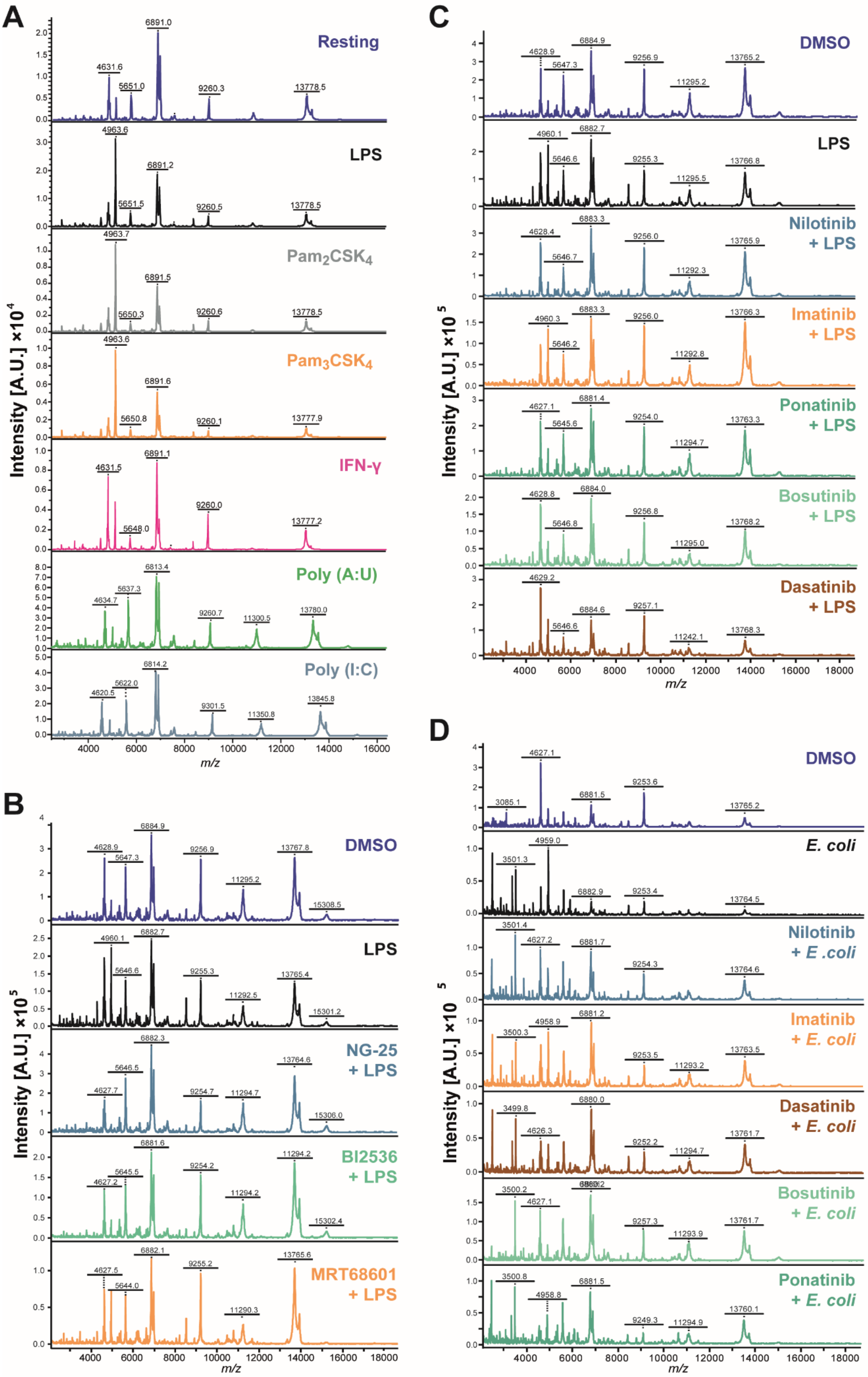

A
Resting
LPS
Pam2CSK4
Pam3CSK4
IFN-γ
Poly (A:U)
Poly (I:C)
Intensity [A.U.] ×10⁴
m/z
B
DMSO
LPS
NG-25 + LPS
BI2536 + LPS
MRT68601 + LPS
Intensity [A.U.] ×10⁵
m/z
C
DMSO
LPS
Nilotinib + LPS
Imatinib + LPS
Ponatinib + LPS
Bosutinib + LPS
Dasatinib + LPS
Intensity [A.U.] ×10⁵
m/z
D
DMSO
E. coli
Nilotinib + E .coli
Imatinib + E. coli
Dasatinib + E. coli
Bosutinib + E. coli
Ponatinib + E. coli
Intensity [A.U.] ×10⁵
m/z

Figure S1

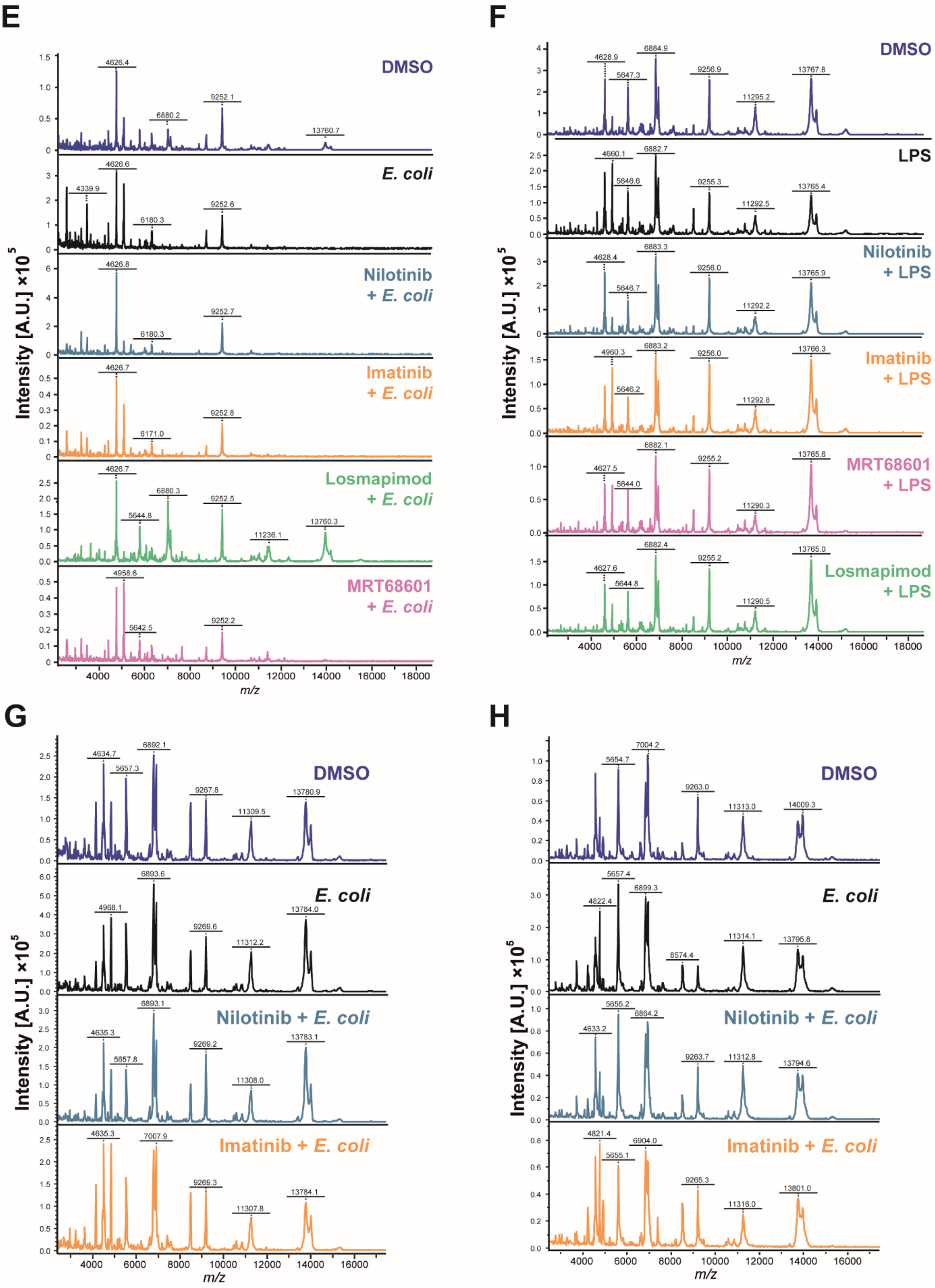
E
DMSO
E. coli
Nilotinib + E. coli
Imatinib + E. coli
Losmapimod + E. coli
MRT68601 + E. coli
Intensity [A.U.] ×10^5
m/z
F
DMSO
LPS
Nilotinib + LPS
Imatinib + LPS
MRT68601 + LPS
Losmapimod + LPS
Intensity [A.U.] ×10^5
m/z
G
DMSO
E. coli
Nilotinib + E. coli
Imatinib + E. coli
Intensity [A.U.] ×10^5
m/z
H
DMSO
E. coli
Nilotinib + E. coli
Imatinib + E. coli
Intensity [A.U.] ×10^5
m/z

Figure S1

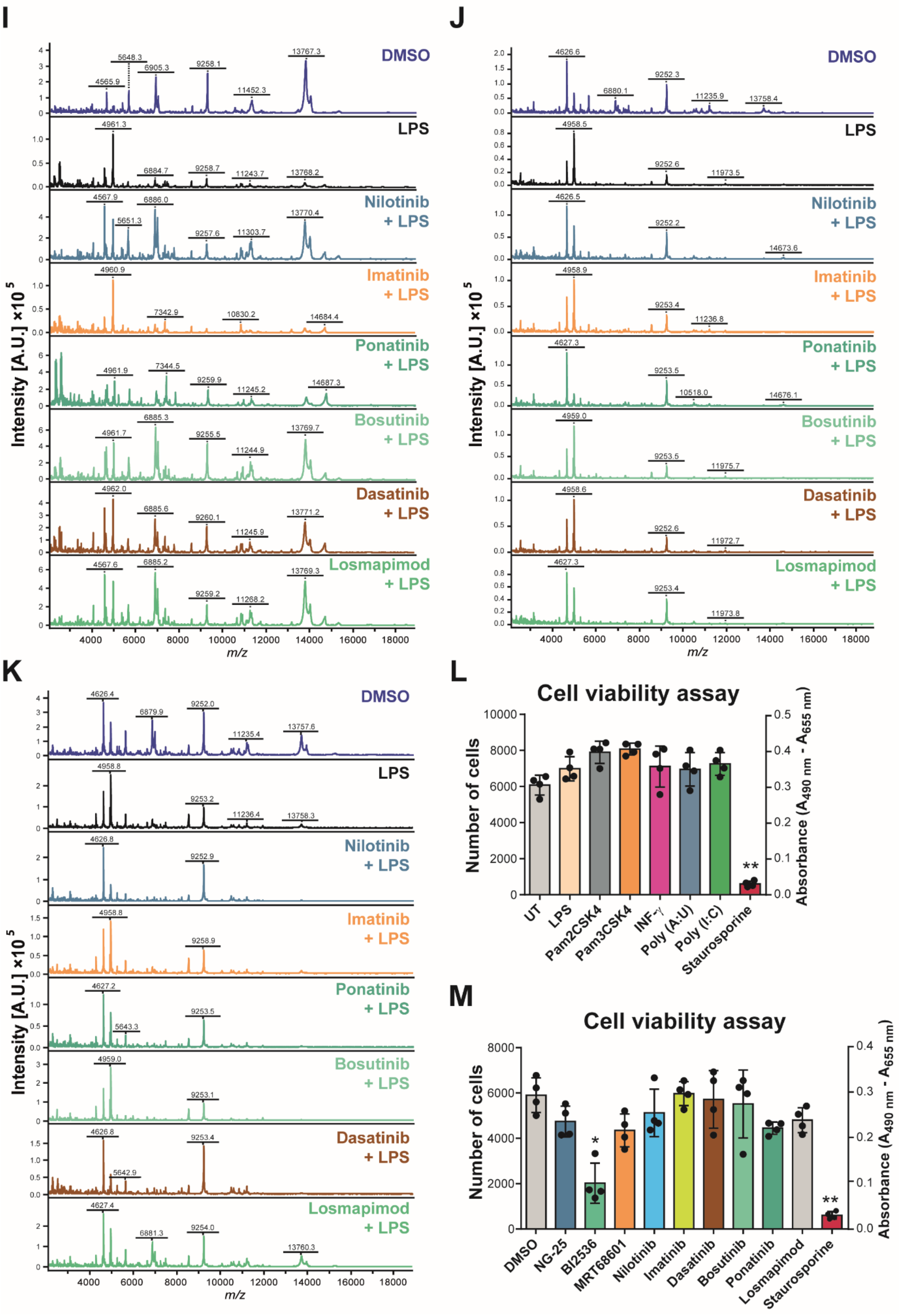
I
J
K
L
M
DMSO
LPS
Nilotinib + LPS
Imatinib + LPS
Ponatinib + LPS
Bosutinib + LPS
Dasatinib + LPS
Losmapimod + LPS
Intensity [A.U.] ×10^5
m/z
Cell viability assay
Number of cells
Absorbance (A490 nm - A655 nm)
UT
LPS
Pam2CSK4
Pam3CSK4
INF-γ
Poly (A:U)
Poly (I:C)
Staurosporine
DMSO
NG-25
BI2536
MRT68601
Nilotinib
Imatinib
Dasatinib
Bosutinib
Ponatinib
Losmapimod
Staurosporine

Figure S1. MALDI-TOF MS spectra. A) Representative MALDI-TOF MS spectra of untreated (resting) THP-1 cells and treated with 100 ng/mL LPS, 100 ng/mL Pam_2CSK_4, 100 ng/mL Pam_3CSK_4, 100 U/mL IFN-γ, 1 μg/mL poly(I:C) or 1 μg/mL poly(A:U) for 24 h. **B)** Representative MALDI-TOF MS spectra of THP-1 cells pre-treated with vehicle control (DMSO), 5 μM NG-25, 5 μM BI2536 or 1 μM MRT68601 for one hour before 100 ng/mL LPS-treatment for up to 24 h. **C-D)** Representative MALDI-TOF MS spectra of THP-1 cells pre-treated with DMSO, 5 μM nilotinib, 5 μM imatinib, 1 μM dasatinib, 1 μM bosutinib or 1 μM ponatinib C) for one hour before 100 ng/mL LPS-treatment for up to 24 h; D) for one hour before stimulation with live *E. coli* for up to 24 h. **E-F)** Representative MALDI-TOF MS spectra of THP-1 cells pre-treated with DMSO, 5 μM nilotinib, 5 μM imatinib, 1 μM losmapimod or 1 μM MRT68601 E) for one hour before stimulation with live *E. coli* for up to 24 h; F) for one hour before 100 ng/mL LPS-treatment for up to 24 h. **G-H)** Representative MALDI-TOF MS spectra of G) OCI-AML2 and H) NCI-H929 cells pre-treated with DMSO, 5 μM nilotinib or imatinib for one hour before stimulation with live *E. coli* for up to 24 h. **I-K)** Representative MALDI-TOF MS spectra of I) primary monocytes and J-K) primary AML cells pre-treated with DMSO, 5 μM nilotinib, 5 μM imatinib, 1 μM dasatinib, 1 μM bosutinib, 1 μM ponatinib or 1 μM losmapimod for one hour before 100 ng/mL LPS-treatment for up to 24 h. **L** and **M**) Represent XTT cell viability assays. Significant differences between two groups were determined by Mann-Whitney U-test. The statistical significance of the comparisons with resting is indicated as follows: **, $P \leq 0.01$; *, $P \leq 0.05$. Error bars represent the standard deviation of four biological replicates.

Figure S2

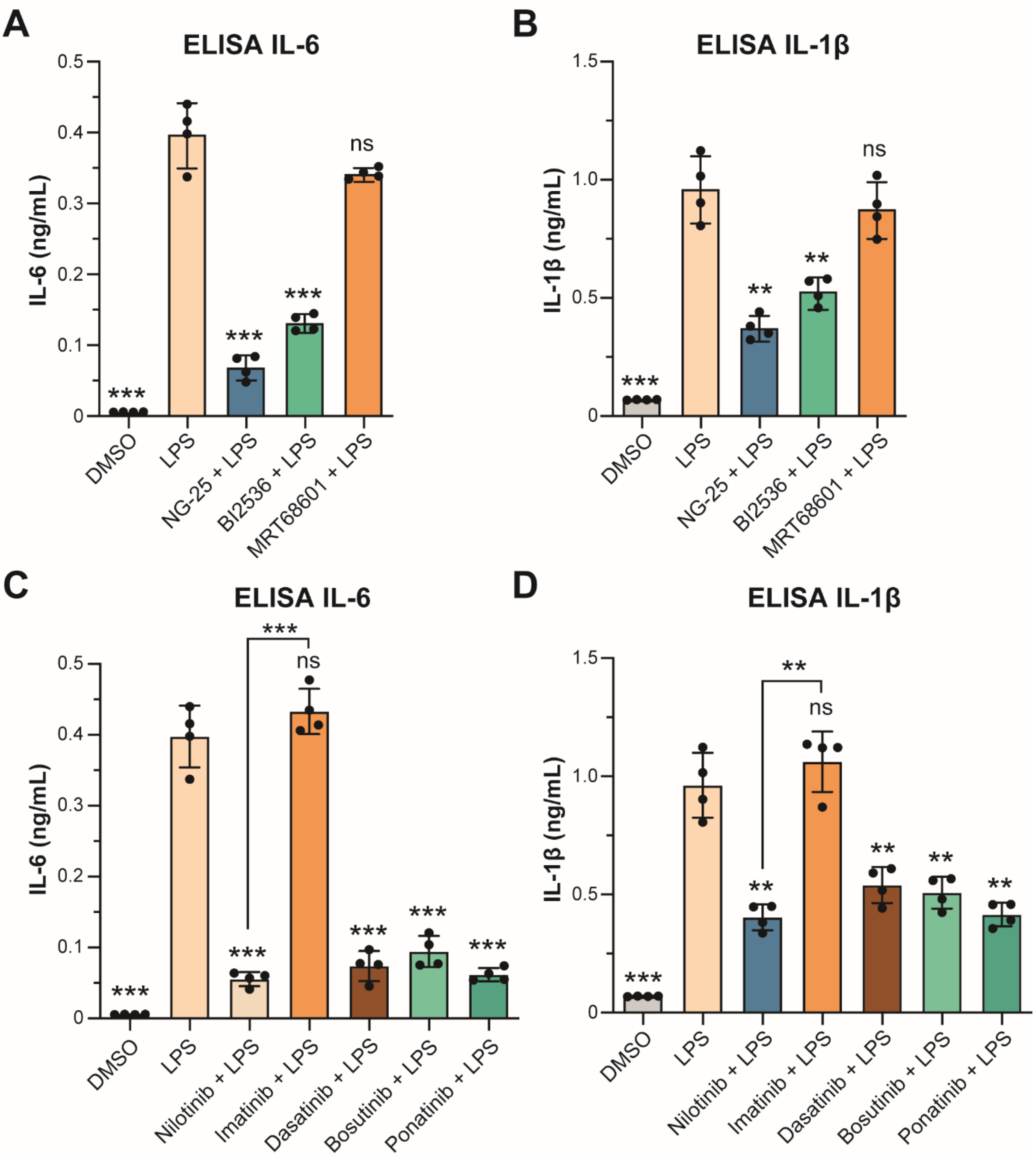

Figure S2. Nilotinib reduces IL-6 and IL-1β secretion. A) IL-6 and **B)** IL-1β secretion of THP-1 cells treated in either vehicle control-treated (DMSO), 100 ng/mL LPS-treated, or pre-treated with 5 µM NG-25, BI2536 or 1 µM MRT68601 for one hour before 100 ng/mL LPS-treatment for up to 24 h measured by ELISA. **C)** IL-6 and **D)** IL-1β secretion of in THP-1 cells in vehicle control-treated (DMSO), 100 ng/mL LPS-treated, or pre-treated with 5 µM nilotinib, imatinib, 1 µM dasatinib, bosutinib or ponatinib for one hour before 100 ng/mL LPS-treatment for up to 24 h measured by ELISA. Significant differences between two groups were determined by a Mann-Whitney U-test. The statistical significance of the comparisons with LPS is indicated as follows: ns, not significant; **, $P \leq 0.01$; ***, $P \leq 0.001$. Error bars represent the standard deviation of four biological replicates.

Figure S3

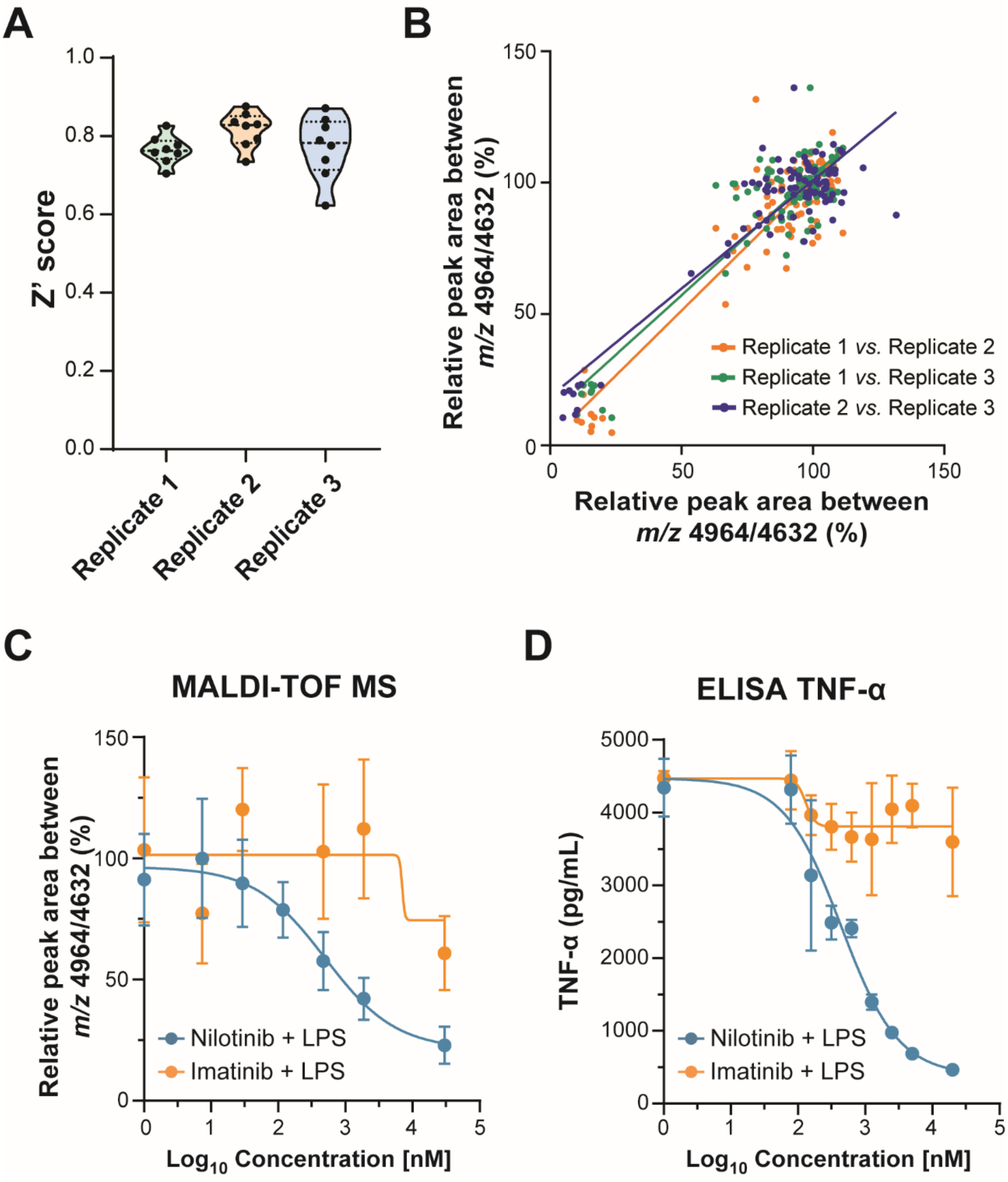

	Nilotinib + LPS
IC50 nM	496.9
R^2	0.7704

	Nilotinib + LPS
IC50 nM	440.8
R^2	0.9121

Figure S3. MALDI-TOF MS screening quality control. A) Z' scores of biological replicates 1, 2 and 3 from the 96 compounds screened across the 8 staggered sets. **B)** Correlation plots of biological replicates 1, 2 and 3 showing correlation $R^2 > 0.80$ and $P \leq 0.001$. **C)** MALDI-TOF MS IC50 curve of nilotinib- and imatinib-pre-treated cells one hour before 100 ng/mL LPS-treatment for up to 24 h. **D)** TNF-α secretion IC50 curve of nilotinib- and imatinib-treated THP-1 cells. Table below shows IC50 concentration (nM) and R^2. Error bars represent the SEM of six (**C**) or four (**D**) biological replicates.

Figure S4

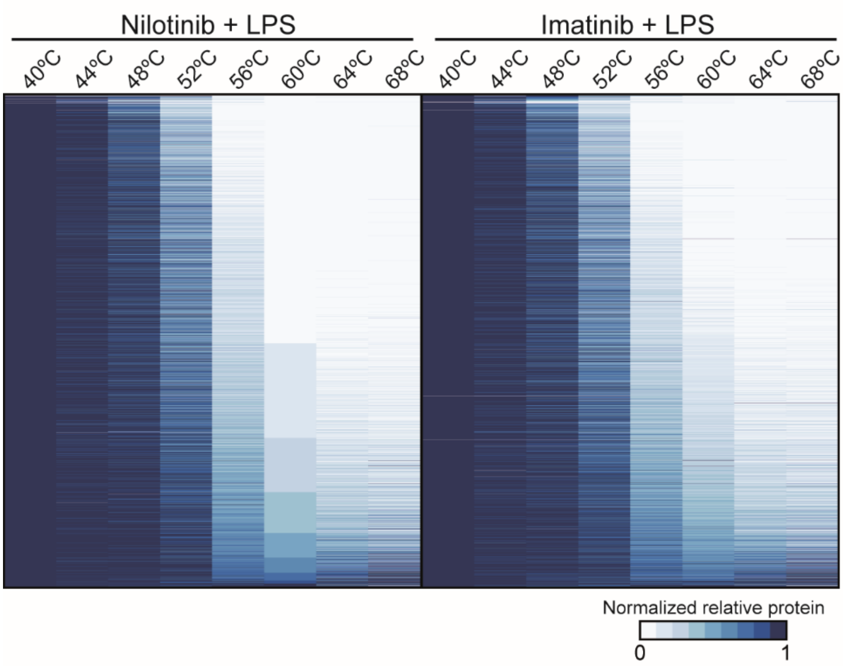

Figure S4. TPP ratio heatmap. Heat map of protein thermal stabilities in THP-1 cells with 5 µM nilotinib and imatinib for one hour before 100 ng/mL LPS-treatment for up to 15 min. The median relative abundance across replicates (n = 4) at the indicated temperature is shown for each protein as fold change relative to the lowest measured temperature (40 °C).

Figure S5

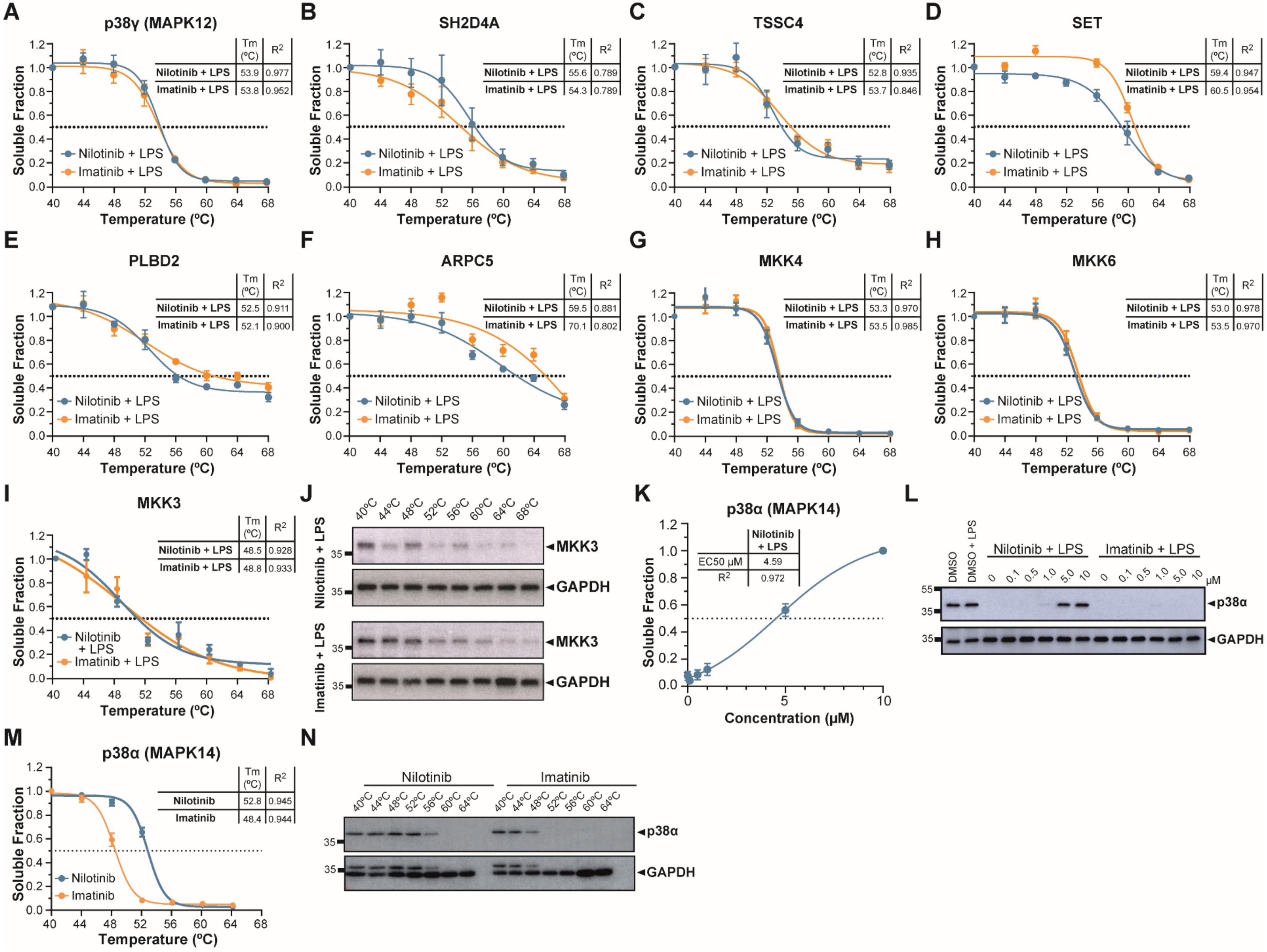
A
p38γ (MAPK12)
Tm (ºC) | R2
Nilotinib + LPS 53.9 0.977
Imatinib + LPS 53.8 0.952
B
SH2D4A
Nilotinib + LPS 55.6 0.789
Imatinib + LPS 54.3 0.789
C
TSSC4
Nilotinib + LPS 52.8 0.935
Imatinib + LPS 53.7 0.846
D
SET
Nilotinib + LPS 59.4 0.947
Imatinib + LPS 60.5 0.954
E
PLBD2
Nilotinib + LPS 52.5 0.911
Imatinib + LPS 52.1 0.900
F
ARPC5
Nilotinib + LPS 59.5 0.881
Imatinib + LPS 70.1 0.802
G
MKK4
Nilotinib + LPS 53.3 0.970
Imatinib + LPS 53.5 0.985
H
MKK6
Nilotinib + LPS 53.0 0.978
Imatinib + LPS 53.5 0.970
I
MKK3
Nilotinib + LPS 48.5 0.928
Imatinib + LPS 48.8 0.933
Soluble Fraction
Temperature (ºC)
Nilotinib + LPS
Imatinib + LPS
J
40ºC 44ºC 48ºC 52ºC 56ºC 60ºC 64ºC 68ºC
Nilotinib + LPS
Imatinib + LPS
MKK3
GAPDH
35
K
p38α (MAPK14)
Nilotinib + LPS
EC50 μM 4.59
R2 0.972
Concentration (μM)
L
DMSO
DMSO + LPS
Nilotinib + LPS
Imatinib + LPS
0 0.1 0.5 1.0 5.0 10 μM
55
35
p38α
GAPDH
M
p38α (MAPK14)
Nilotinib 52.8 0.945
Imatinib 48.4 0.944
Nilotinib
Imatinib
N
Nilotinib
Imatinib
40ºC 44ºC 48ºC 52ºC 56ºC 60ºC 64ºC
p38α
GAPDH

Figure S5. TPP analysis. A) Determination by TPP analysis of the thermostability of p38γ (MAPK12), **B)** SH2 domain-containing protein 4A (SH2D4A), **C)** protein TSSC4, **D)** protein SET, **E)** putative phospholipase B-like 2 (PLBD2), **F)** Actin-related protein 2/3 complex subunit 5 (ARPC5), **G)** Mitogen-activated protein kinase kinase 4 (MKK4), **H)** Mitogen-activated protein kinase kinase 6 (MKK6) protein at the indicated temperatures with 5 µM nilotinib or imatinib for one hour before 100 ng/mL LPS-treatment for up to 15 min. Table insert shows melting temperature (Tm ºC) and R^2. **I-J)** Determination of the thermostability of MKK3 at the indicated temperatures in THP-1 cells pre-treated with 5 µM nilotinib or imatinib for one hour before 100 ng/mL LPS-treatment for up to 15 min. J) Representative western blots of the thermostability of MKK3. GAPDH served as a loading control. Table insert shows melting temperature (Tm ºC) and R^2. **K-L)** Determination of the thermostability of p38α (MAPK14) at 56ºC in DMSO, 100 ng/mL LPS and pre-treated with the indicated concentrations of nilotinib or imatinib for one hour before 100 ng/mL LPS-treatment for up to 15 min. Table insert below shows effective concentration (EC50) (µM) and R^2. **M-N)** Determination of the thermostability of p38α (MAPK14) at 56ºC in 5 µM nilotinib or imatinib for one hour. Table insert shows melting temperature (Tm ºC) and R^2. Error bars represent the SEM of four biological replicates. GAPDH served as a loading control. A representative image of four replicates is shown. Relative mobilities of reference proteins (masses in kilo Daltons) are shown on the left of each blot.

Figure S6

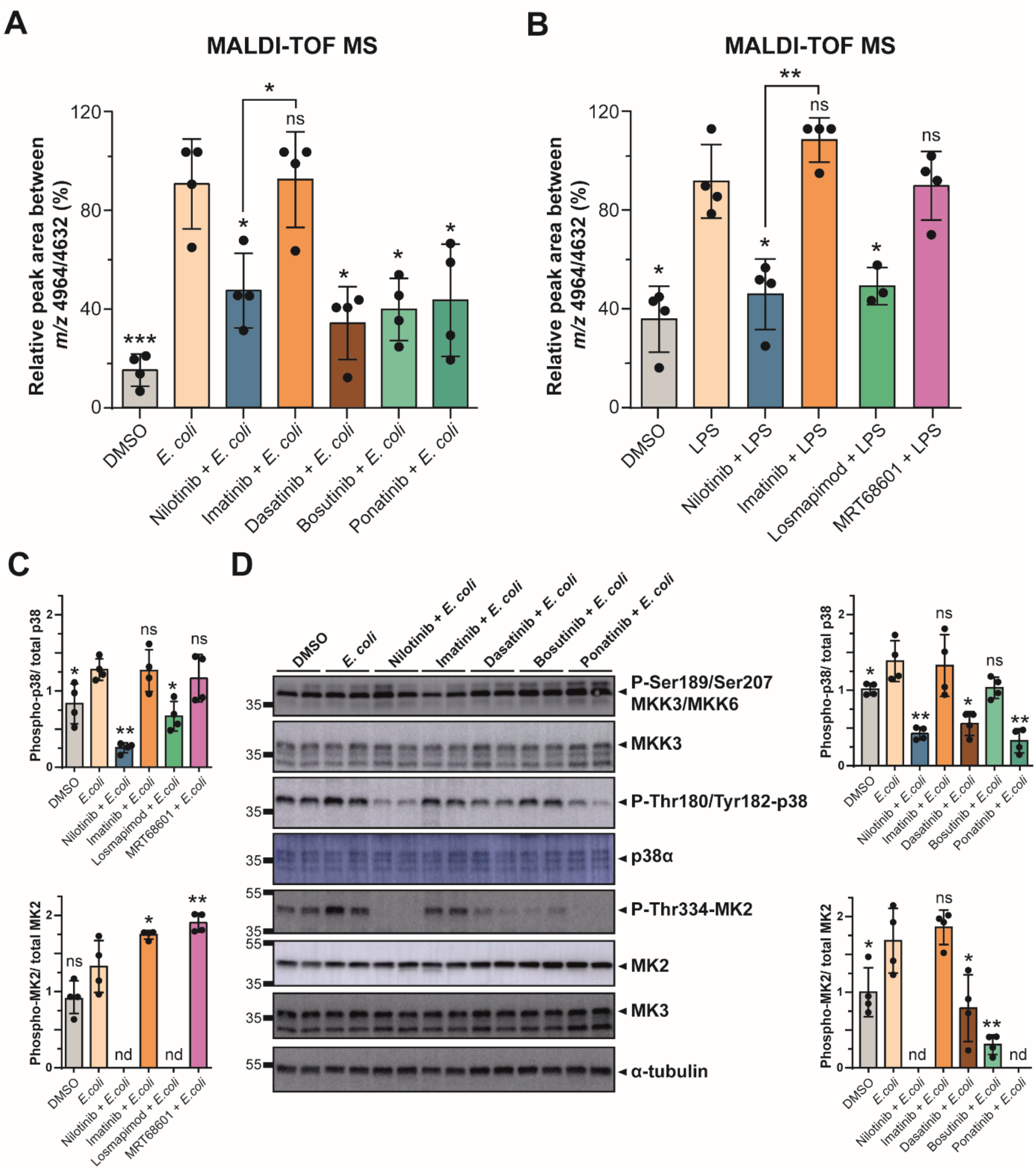

Figure S6. Nilotinib reduces the pro-inflammatory phenotype. A) MALDI-TOF MS relative quantitation of the ratio *m/z* 4964 / 4632 in THP-1 cells pre-treated with DMSO, 5 µM nilotinib, imatinib, 1 µM dasatinib, bosutinib or ponatinib for one hour before stimulation with live *E. coli* for up to 24 h. **B)** MALDI-TOF MS relative quantitation of the ratio *m/z* 4964 / 4632 in THP-1 cells pre-treated with DMSO, 5 µM nilotinib, imatinib, 1 µM losmapimod or MRT68601 for one hour before stimulation with LPS for up to 24 h. **C)** Relative quantification of four replicates (Figure 4B) is shown

on the right for p38α and MK2 phosphorylation levels. **D)** Western blot analysis of the p38α MAPK pathway in THP-1 cells pre-treated with DMSO, 5 µM nilotinib, 5 µM nilotinib, imatinib, 1 µM dasatinib, bosutinib or ponatinib for one hour before stimulation with live *E. coli* for up to 15 min. α-tubulin serves as a loading control. A representative image with two biological replicates of four replicates is shown. Relative mobilities of reference proteins (masses in kilo Daltons) are shown on the left of each blot. Relative quantification is shown on the right for p38α and MK2 phosphorylation levels. Error bars represent the standard deviation of four biological replicates. Significant differences between two groups were determined by a Mann-Whitney U-test. The statistical significance of the comparisons with *E. coli* is indicated as follows: ns, not significant; nd, not detected; *, $P \leq 0.05$; **, $P \leq 0.01$; ***, $P \leq 0.001$.

Figure S7

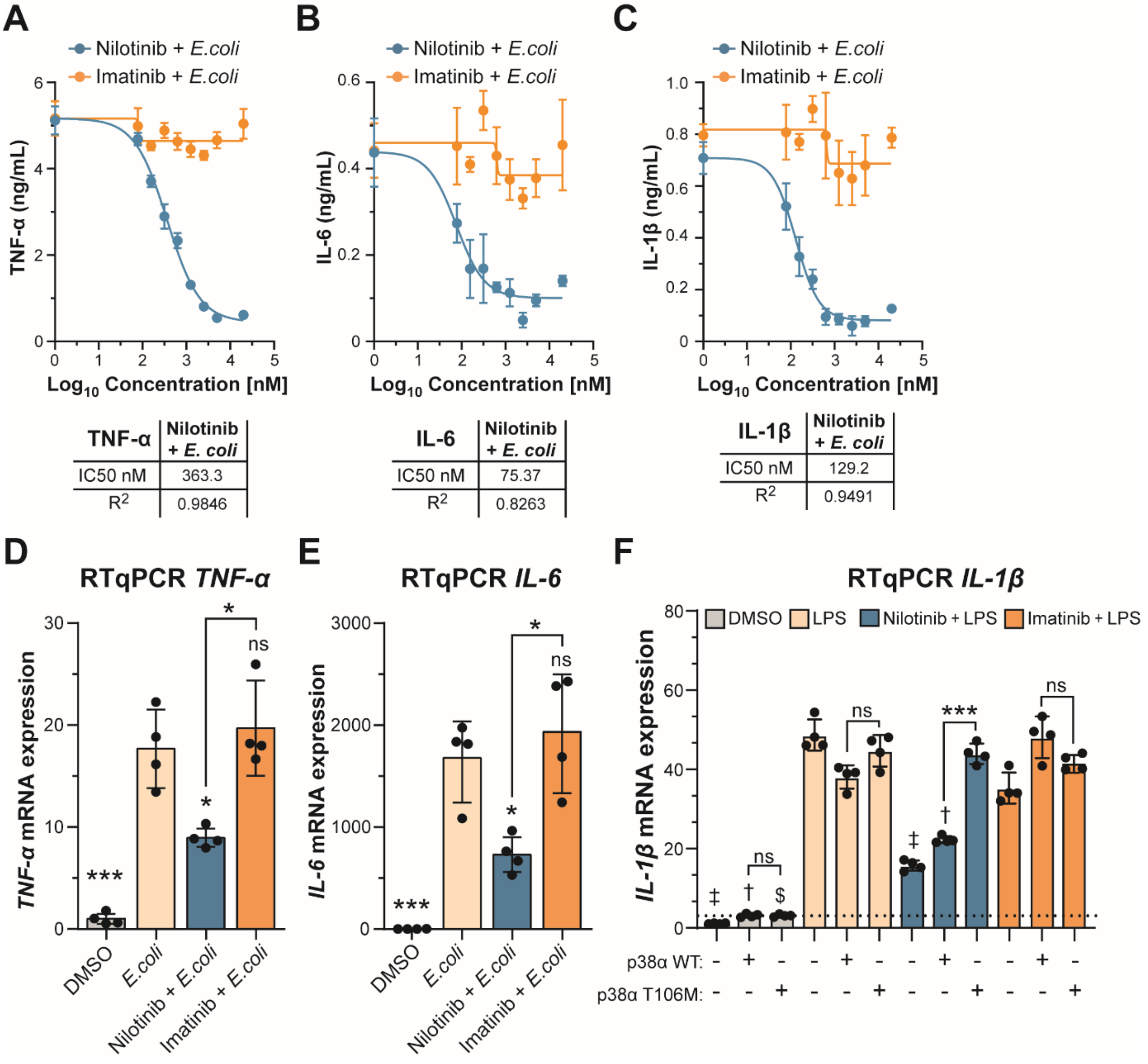

Figure S7. Nilotinib inhibits the pro-inflammatory phenotype in *E. coli*-activated monocytes. A-C) ELISA IC50 curves of A) TNF-α, B) IL-6, and C) IL-1β were measured by ELISA at 24 h. Error bars represent the standard deviation of four biological replicates. Significant differences between two groups were determined by a Students t-test. The statistical significance of the comparisons with *E. coli* is indicated as follows: ns, not significant; **, $P \leq 0.01$; ***, $P \leq 0.001$. Table insert shows IC50 (nM) and R^2. **D)** *TNF-α*, and **E)** *IL-6* expression were determined by RT-qPCR in THP-1 cells pre-treated with DMSO, 5 µM nilotinib or imatinib for one hour before stimulation with live *E. coli* for up to 24 h. The results were analyzed using the $2^{-\Delta\Delta Ct}$ method and normalized using *GAPDH* and *TBP* as the reference genes; and DMSO as the reference sample. Error bars represent the standard deviation of four biological replicates. The statistical significance of the comparisons with *E. coli* is indicated as follows: ns, not significant; **, $P \leq 0.01$; ***, $P \leq 0.001$. **F)** *IL-6* expression was determined by RT-qPCR in non-transfected or transfected HEK293 cells with p38α WT or p38α M107T for 48 h and pre-treated with DMSO, 5 µM nilotinib or imatinib for one hour before stimulation with LPS for up to 24 h. The results were analyzed using the $2^{-\Delta\Delta Ct}$ method and normalized using *GAPDH* and *TBP* as the reference genes; and DMSO as the reference sample. The statistical significance of the comparisons with: LPS non-transfected is indicated as ‡; LPS transfected with p38α WT is indicated as †; LPS transfected with p38α T106M is indicated as $. The statistical significance of the comparisons between p38α WT and p38α M107T is indicated as follows: ns, not significant; ***, $P \leq 0.001$. Error bars represent the standard deviation of four biological replicates. Significant differences between two groups were determined by Mann-Whitney U-test.

Figure S8

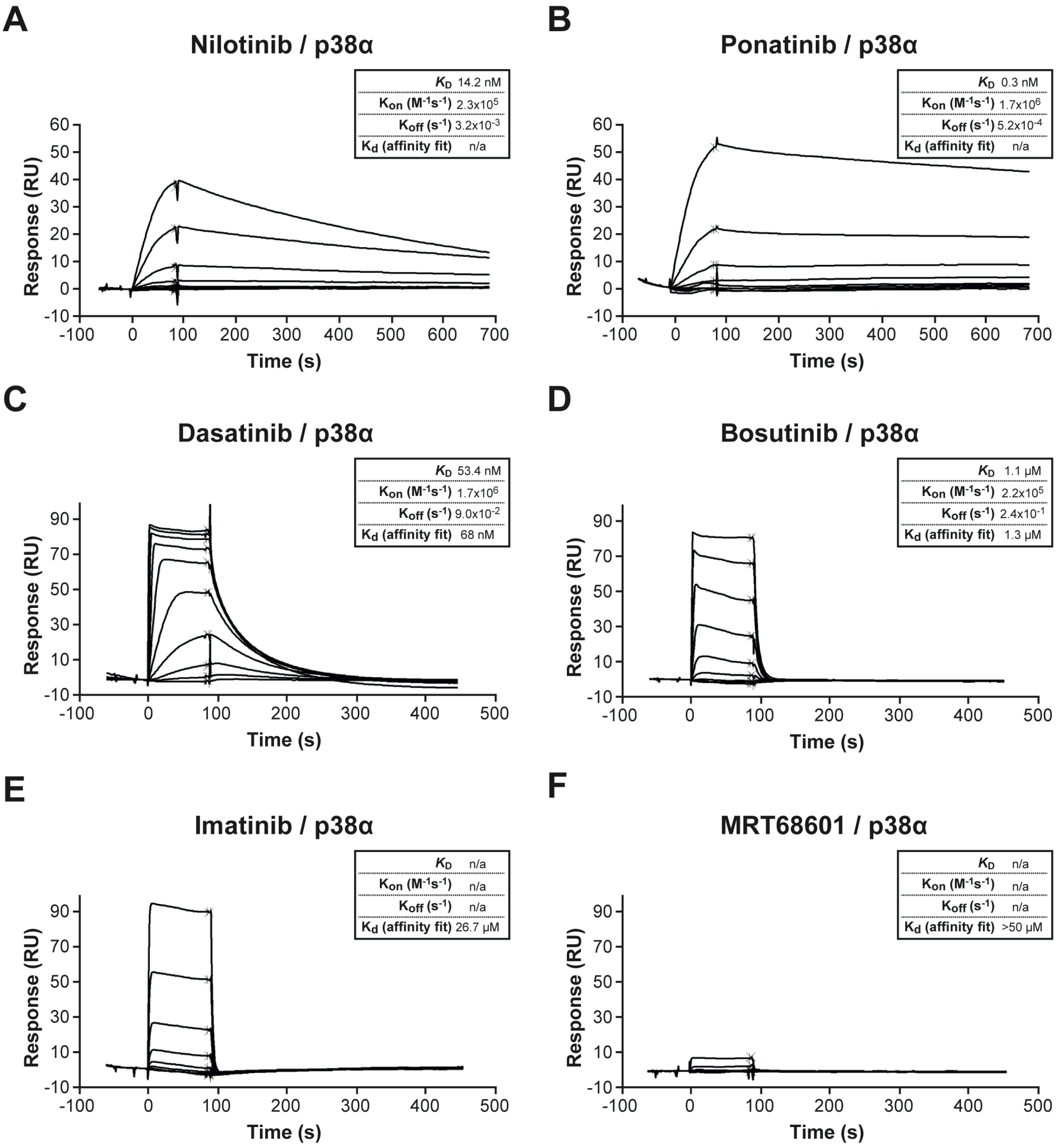

Figure S8. Assessment of TKIs against p38α using surface plasmon resonance (SPR). (A-G) Representative multicycle SPR sensograms for **A)** nilotinib, **B)** ponatinib, **C)** dasatinib, **D)** bosutinib, **E)** imatinib, and **F)** MRT68601showing a dose-dependent concentration series against immobilized p38α. K_D, equilibrium dissociation rate constant; K_{on} (M^{-1} s^{-1}), on-rate constant or association reaction; K_{off} (s^{-1}), off-rate constant or dissociation reaction; Kd (affinity fit), dissociation rate constant. Experiments performed in triplicate.

Figure S9

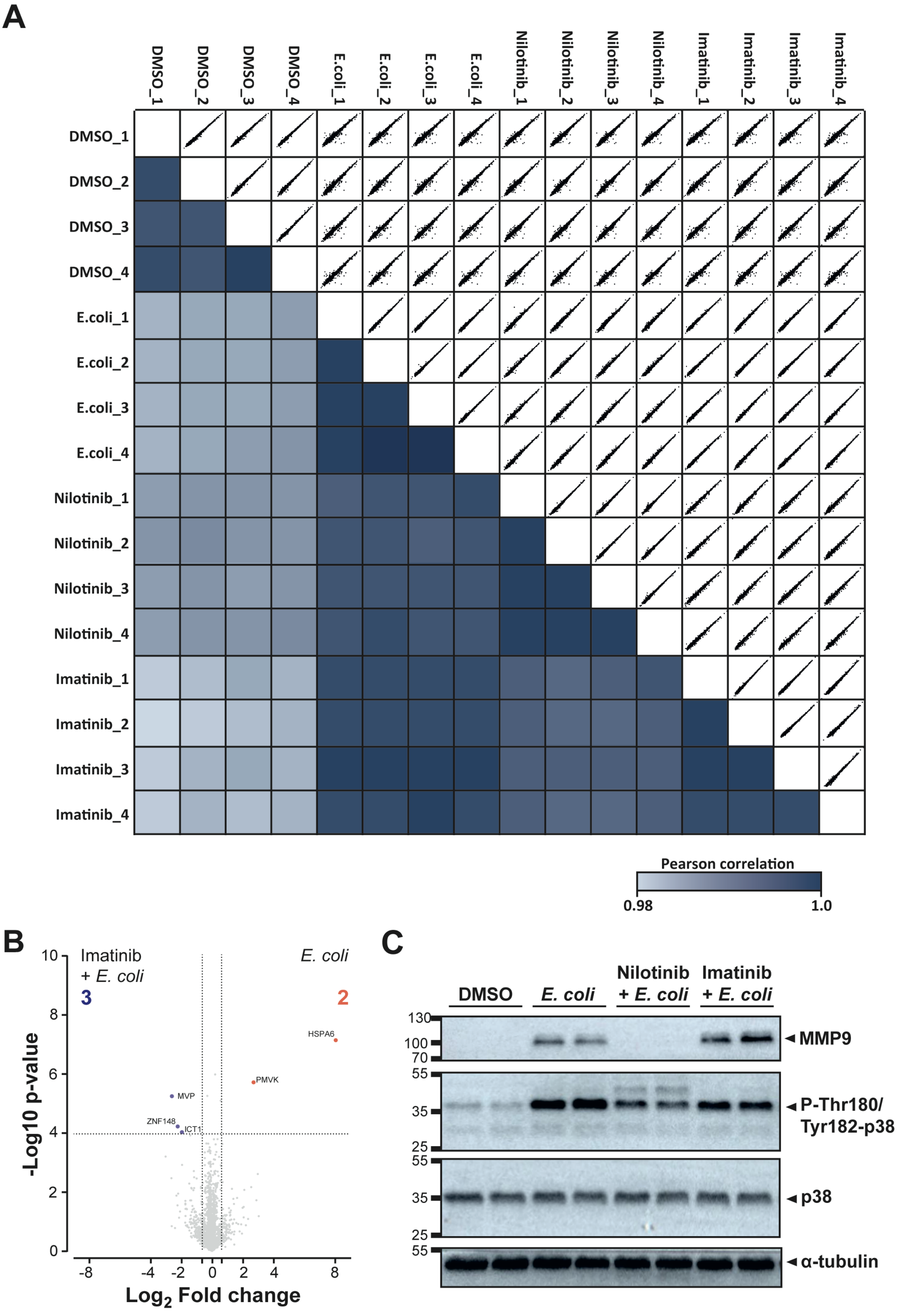
A
DMSO_1
DMSO_2
DMSO_3
DMSO_4
E.coli_1
E.coli_2
E.coli_3
E.coli_4
Nilotinib_1
Nilotinib_2
Nilotinib_3
Nilotinib_4
Imatinib_1
Imatinib_2
Imatinib_3
Imatinib_4
Pearson correlation
0.98
1.0
B
Imatinib + E. coli
3
E. coli
2
HSPA6
PMVK
MVP
ZNF148
ICT1
-Log10 p-value
Log2 Fold change
C
DMSO
E. coli
Nilotinib + E. coli
Imatinib + E. coli
130
100
70
MMP9
55
35
25
P-Thr180/ Tyr182-p38
55
35
25
p38
55
α-tubulin

Figure S9. Differences in the proteome of nilotinib- and imatinib-treated monocytes. A) The matrix of correlation plots revealed very high correlations between log_2 transformed LFQ intensities (Pearson correlation coefficient between 0.982 and 0.998). The color code indicates the values of correlation coefficients. **B)** Volcano plot of THP-1 cells treated with 5 μM nilotinib *vs. E. coli*, cut-off of FDR <0.05 and 1.5-fold change between conditions. **C)** MMP9 and p38α levels in THP-1 pre-treated with 5 μM nilotinib or imatinib for one hour before stimulation with live *E. coli* for up to 24 h. α-tubulin serves as a loading control. A representative image with two biological replicates of four replicates is shown. Relative mobilities of reference proteins (masses in kilo Daltons) are shown on the left of each blot.

Figure S10

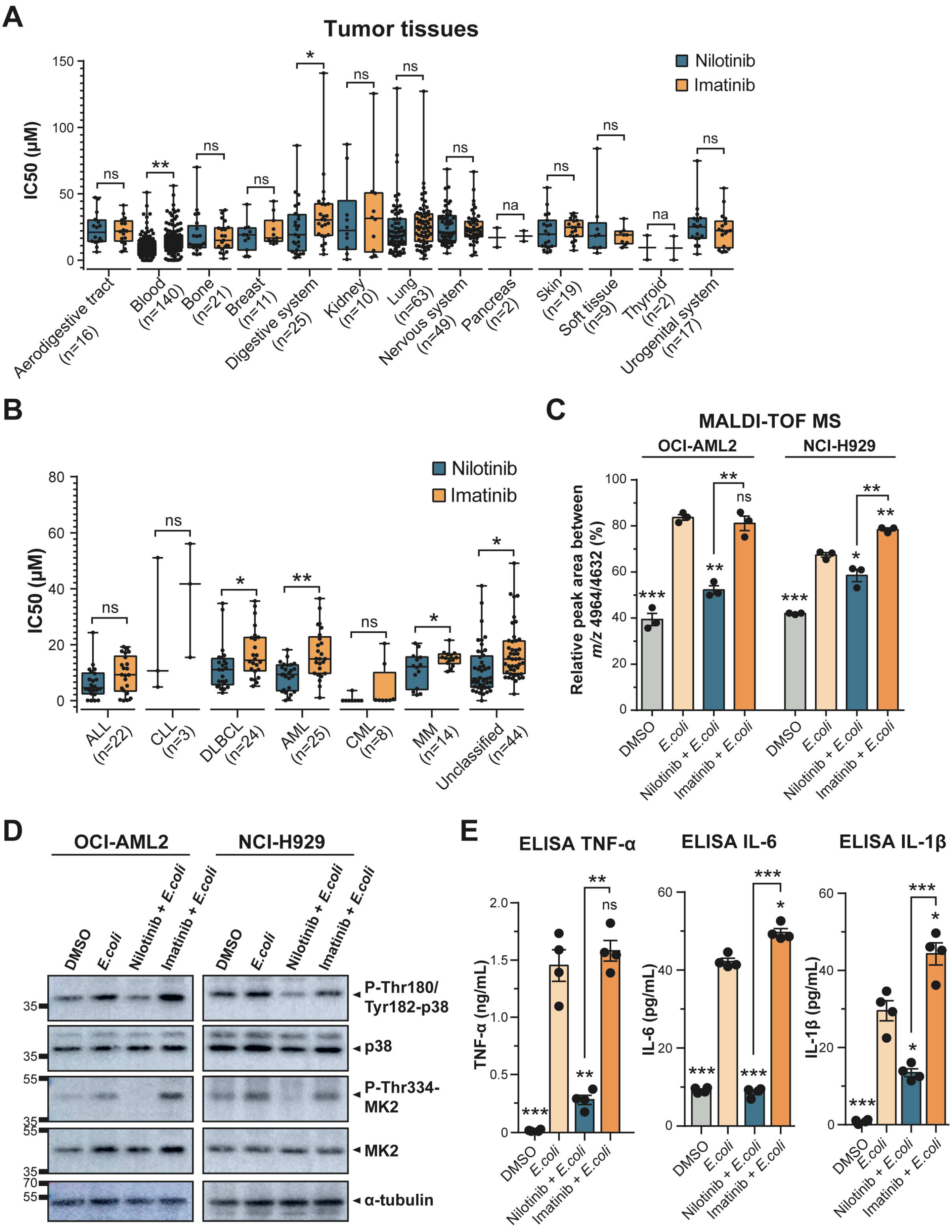
A
Tumor tissues
IC50 (μM)
Nilotinib
Imatinib
Aerodigestive tract (n=16)
Blood (n=140)
Bone (n=21)
Breast (n=11)
Digestive system (n=25)
Kidney (n=10)
Lung (n=63)
Nervous system (n=49)
Pancreas (n=2)
Skin (n=19)
Soft tissue (n=9)
Thyroid (n=2)
Urogenital system (n=17)
B
ALL (n=22)
CLL (n=3)
DLBCL (n=24)
AML (n=25)
CML (n=8)
MM (n=14)
Unclassified (n=44)
C
MALDI-TOF MS
OCI-AML2
NCI-H929
Relative peak area between m/z 4964/4632 (%)
DMSO
E.coli
Nilotinib + E.coli
Imatinib + E.coli
D
OCI-AML2
NCI-H929
P-Thr180/ Tyr182-p38
p38
P-Thr334-MK2
MK2
α-tubulin
E
ELISA TNF-α
ELISA IL-6
ELISA IL-1β
TNF-α (ng/mL)
IL-6 (pg/mL)
IL-1β (pg/mL)

Figure S10. Nilotinib and imatinib show different sensitivity in blood malignancies. Nilotinib reduces the inflammation phenotype in OCI-AML2 and NCI-H929 cell line. A) IC50 values of nilotinib and imatinib in various types of cancer, and **B)** in hematological malignancies according to TCGA classification from the Genomics of Drug Sensitivity in Cancer (GDSC) database. **C)** MALDI-TOF MS relative quantitation of the ratio m/z 4964/4632 in NCI-H929 cells pre-treated with DMSO, 5 μM nilotinib or imatinib for one hour before stimulation with live E. coli for up to 24 h. **D)** p38α MAPK pathway status in NCI-H929 cells. α-tubulin was detected as a loading control. A representative image of four replicates is shown. Relative mobilities of reference proteins (masses in kilo Daltons) are shown on the left of each blot. **E)** TNF-α, IL-6, and IL-1β secretion was measured by ELISA at 24 h. Error bars represent the SEM of four biological replicates. Significant differences between two groups were determined by a Mann-Whitney U-test. The statistical significance of the comparisons with *E. coli* is indicated as follows: ns, not significant; na, not applicable; *, $P \leq 0.05$; **, $P \leq 0.01$; ***, $P \leq 0.001$.
